# Supplementary material for: Adenosine-to-inosine editing of endogenous Z-form RNA by the deaminase ADAR1 prevents spontaneous MAVS-dependent type I interferon responses
Source: Immunity. 2021 Sep 14;54(9):1961–1975.e5. doi: 10.1016/j.immuni.2021.08.011 (PMC8459395; doi:10.1016/j.immuni.2021.08.011)
Supplement: Document S1. Figures S1–S7 and Table S2 [file mmc1.pdf]

Immunity, Volume *54*

Supplemental information

Adenosine-to-inosine editing of endogenous Z-form RNA by the deaminase ADAR1 prevents spontaneous MAVS-dependent type I interferon responses

Qiannan Tang, Rachel E. Rigby, George R. Young, Astrid Korning Hvidt, Tanja Davis, Tiong Kit Tan, Anne Bridgeman, Alain R. Townsend, George Kassiotis, and Jan Rehwinkel

Figure S1.

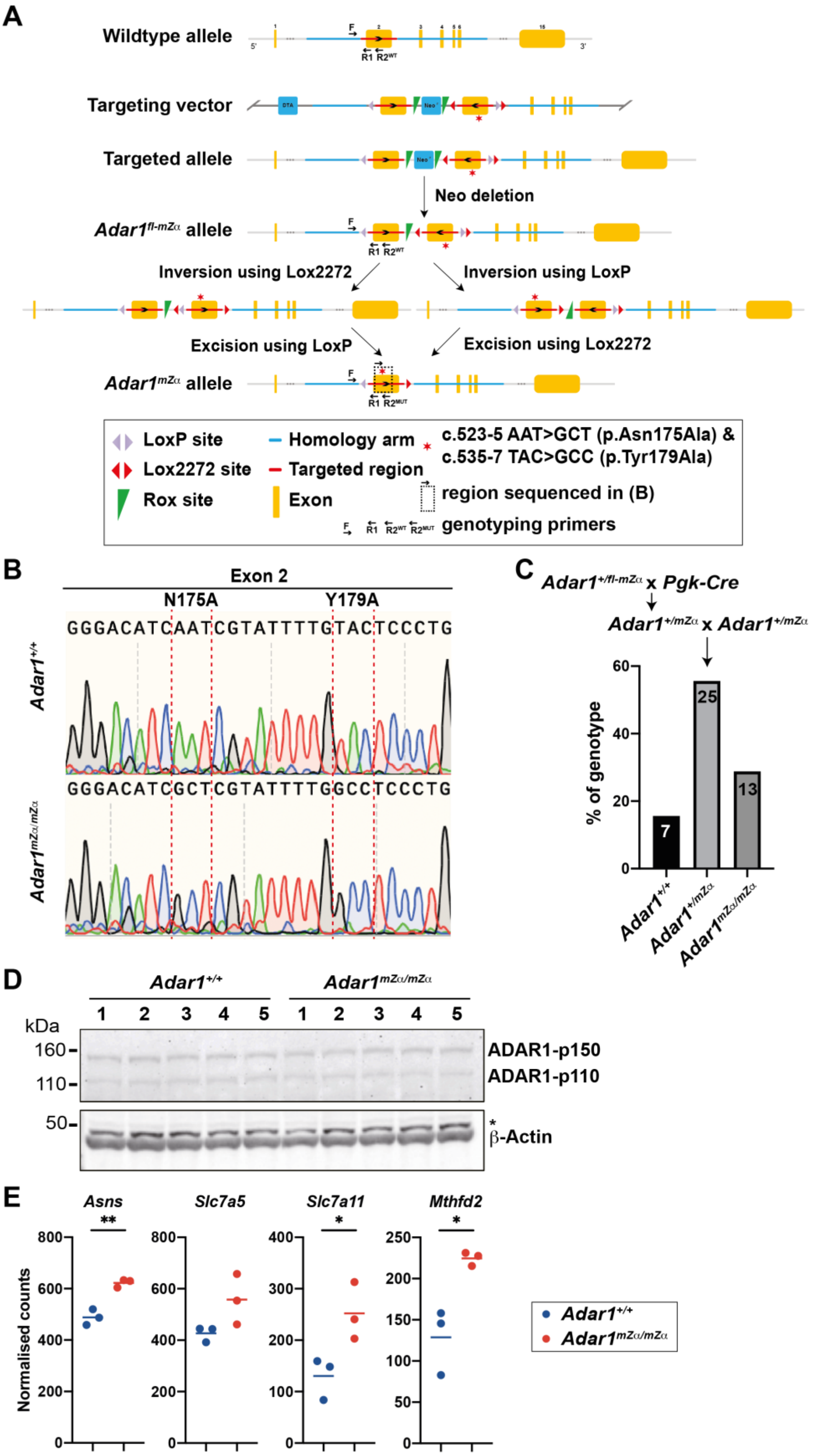

A
Wildtype allele
Targeting vector
Targeted allele
Neo deletion
Adar1fl-mZα allele
Inversion using Lox2272
Inversion using LoxP
Excision using LoxP
Excision using Lox2272
Adar1mZα allele
LoxP site
Lox2272 site
Rox site
Homology arm
Targeted region
Exon
c.523-5 AAT>GCT (p.Asn175Ala) &
c.535-7 TAC>GCC (p.Tyr179Ala)
region sequenced in (B)
genotyping primers
B
Exon 2
N175A
Y179A
GGGACATCAATCGTATTTTGTACTCCCTG
GGGACATCGCTCGTATTTTGGCCTCCCTG
Adar1+/+
Adar1mZα/mZα
C
Adar1+/fl-mZα x Pgk-Cre
Adar1+/mZα x Adar1+/mZα
% of genotype
60
40
20
0
7
25
13
Adar1+/+
Adar1+/mZα
Adar1mZα/mZα
D
Adar1+/+
Adar1mZα/mZα
1 2 3 4 5 1 2 3 4 5
kDa
160
110
50
ADAR1-p150
ADAR1-p110
*
β-Actin
E
Asns
Slc7a5
Slc7a11
Mthfd2
Normalised counts
**
*
*
Adar1+/+
Adar1mZα/mZα

Figure S1, related to Figures 1 and 2. Generation of *Adar1*$^{mZ\alpha/mZ\alpha}$ animals.

A. Schematic representation of the *Adar1* WT allele, targeting vector, targeted allele, *Adar1*$^{fl\text{-}mZ\alpha}$ allele and the two-step Cre-mediated recombination process that resulted in the *Adar1*$^{mZ\alpha}$ allele. Please see text for details.

B. Genomic DNA was prepared from WT and *Adar1*$^{mZ\alpha/mZ\alpha}$ animals and the mutated region in exon 2 was sequenced.

C. *Adar1*$^{+/fl\text{-}mZ\alpha}$ mice were bred with the *Pgk-Cre* line. *Adar*$^{+/mZ\alpha}$ offspring were then mated to generate *Adar1*$^{mZ\alpha/mZ\alpha}$ animals. The numbers and percentages of animals obtained with the indicated genotypes are shown.

D. BMMCs were grown from bone marrow from five mice of the indicated genotypes. Protein extracts were used for western blot with α-ADAR1 antibody. β-Actin served as a loading control. *, non-specific band

E. Normalised read counts for the indicated transcripts from lung RNA sequencing (see Figure 2) are shown.

Figure S2.

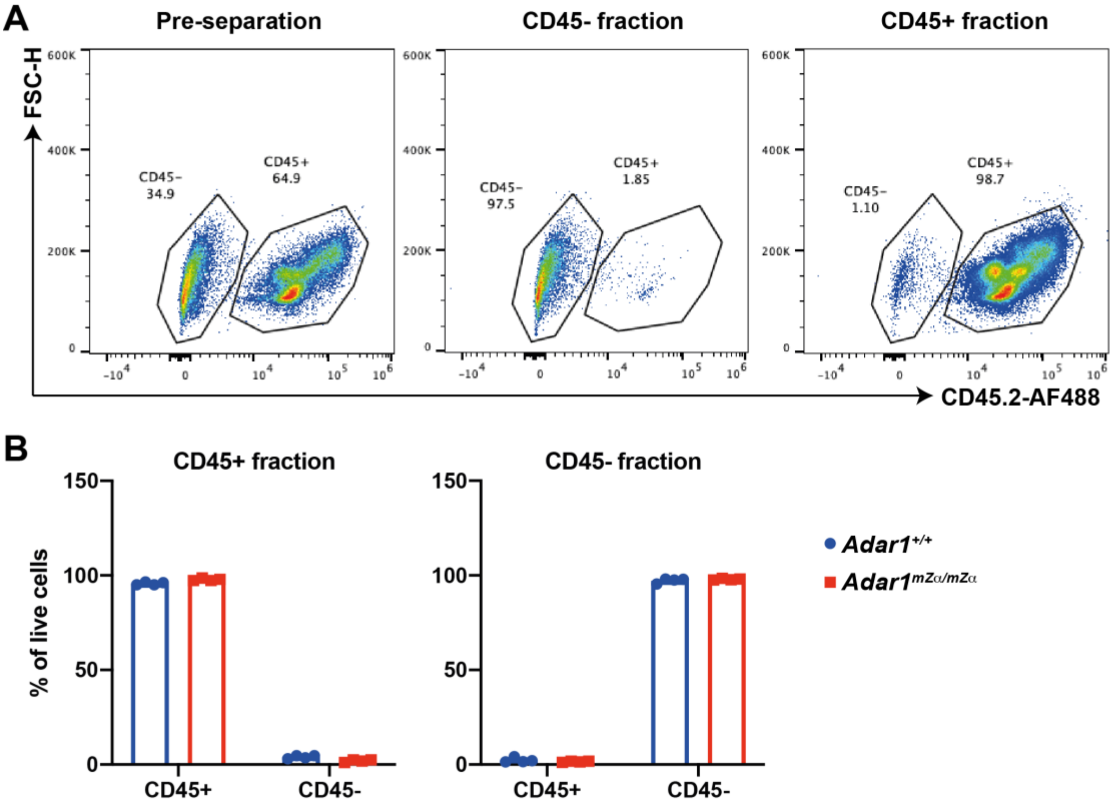

Figure S2, related to Figure 3. MACS separation of lung cells.

A. Cell surface levels of CD45 were analysed by flow cytometry in single cell suspensions obtained from lung tissue before MACS (left) and in CD45- and CD45+ cell fractions obtained after MACS (middle and right). Data are from a representative WT animal.

B. The percentage of CD45-expressing cells is shown for CD45+ and CD45- MACS fractions. Data points represent individual animals (n=4) from a representative experiment and bars indicate the mean.

Figure S3.

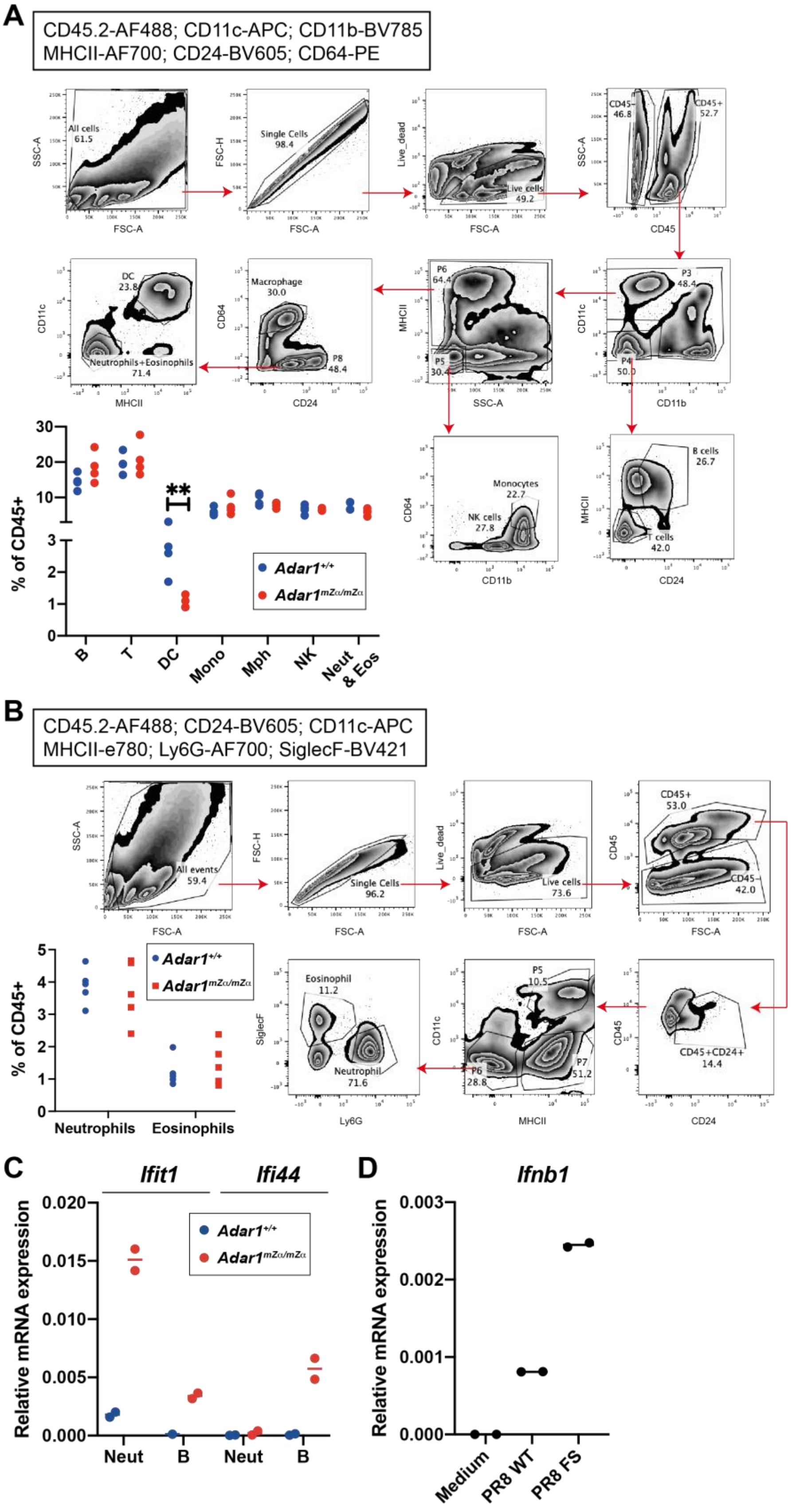
A
CD45.2-AF488; CD11c-APC; CD11b-BV785
MHCII-AF700; CD24-BV605; CD64-PE
All cells 61.5
Single Cells 98.4
Live cells 49.2
CD45- 46.8
CD45+ 52.7
DC 23.8
Neutrophils+Eosinophils 71.4
Macrophage 30.0
P8 48.4
P6 64.4
P5 30.4
P3 48.4
P4 50.0
Monocytes 22.7
NK cells 27.8
B cells 26.7
T cells 42.0
% of CD45+
Adar1+/+
Adar1mZα/mZα
B
T
DC
Mono
Mph
NK
Neut & Eos
B
CD45.2-AF488; CD24-BV605; CD11c-APC
MHCII-e780; Ly6G-AF700; SiglecF-BV421
All events 59.4
Single Cells 96.2
Live cells 73.6
CD45+ 53.0
CD45- 42.0
CD45+CD24+ 14.4
P5 10.5
P7 51.2
P6 28.8
Eosinophil 11.2
Neutrophil 71.6
Neutrophils
Eosinophils
C
Ifit1
Ifi44
Relative mRNA expression
Neut
B
D
Ifnb1
Medium
PR8 WT
PR8 FS

Figure S3, related to Figure 4A and 4B. Gating strategy for sorting of haematopoietic lung cells.

A-B. Two staining panels were used to identify and isolate haematopoietic cell populations by FACS. Panel (A) is related to Figure 4A and panel (B) to Figure 4B. Antibodies and conjugated fluorophores are shown in boxes. Gating strategies are shown for a representative WT (A) and *Adar1*$^{mZ\alpha/mZ\alpha}$ (B) animal. Bar graphs show the proportion of each cell population as a percentage of CD45+ cells. Each dot represents an individual mouse and data from two independent experiments were pooled (**p<0.01, unpaired t test).

C. Neutrophils and B cells were sorted from the lungs of WT and *Adar1*$^{mZ\alpha/mZ\alpha}$ mice. RNA samples were reverse transcribed with gene specific primers for *Ifit1*, *Ifi44* and *Ifnb1*, followed by qPCR. *Ifnb1* mRNA levels were undetectable.

D. RNA extracted from whole lungs of WT mice 48 hours after intranasal infection with 50,000 pfu of IAV PR8 or of a mutant PR8 virus (PR8 FS; known to induce higher levels of *Ifnb1*) were analysed as in (C).

In (C-D), mRNA levels are shown relative to *Actb*. Each dot represents samples from an individual mouse.

Figure S4.

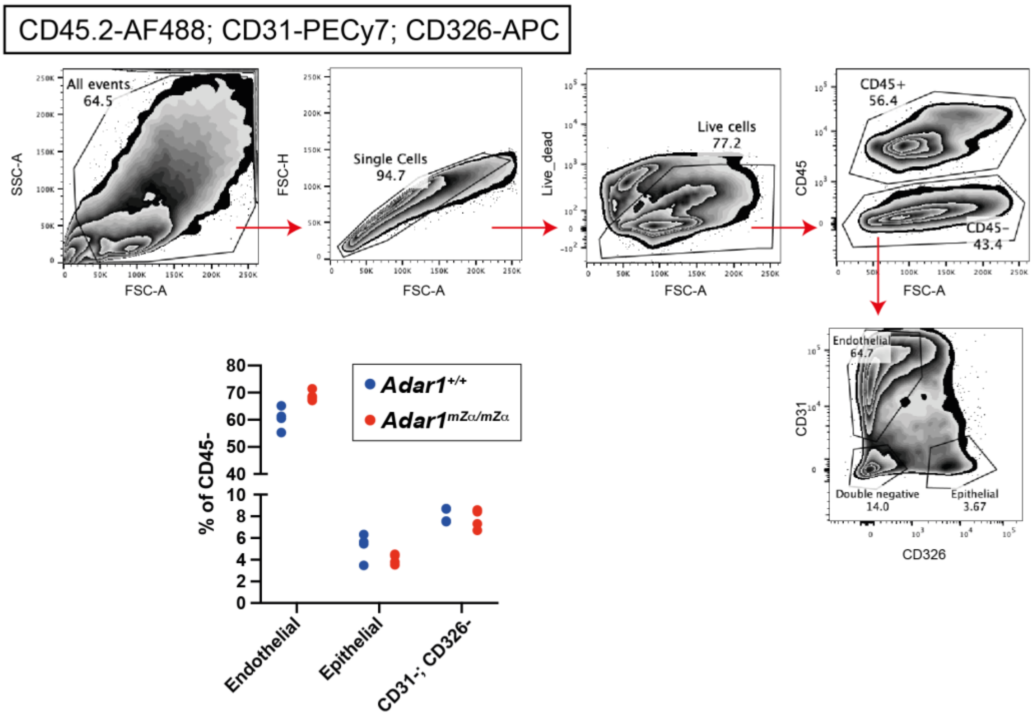

<u>Figure S4, related to Figure 4C. Gating strategy for sorting of stromal lung cells.</u> The staining panel used to identify and isolate non-haematopoietic cell populations by FACS is shown. Antibodies and conjugated fluorophores are shown in the box. The gating strategy is shown for a representative WT animal. The bar graph shows the proportion of each cell population as a percentage of CD45- cells. Each dot represents an individual mouse.

Figure S5.

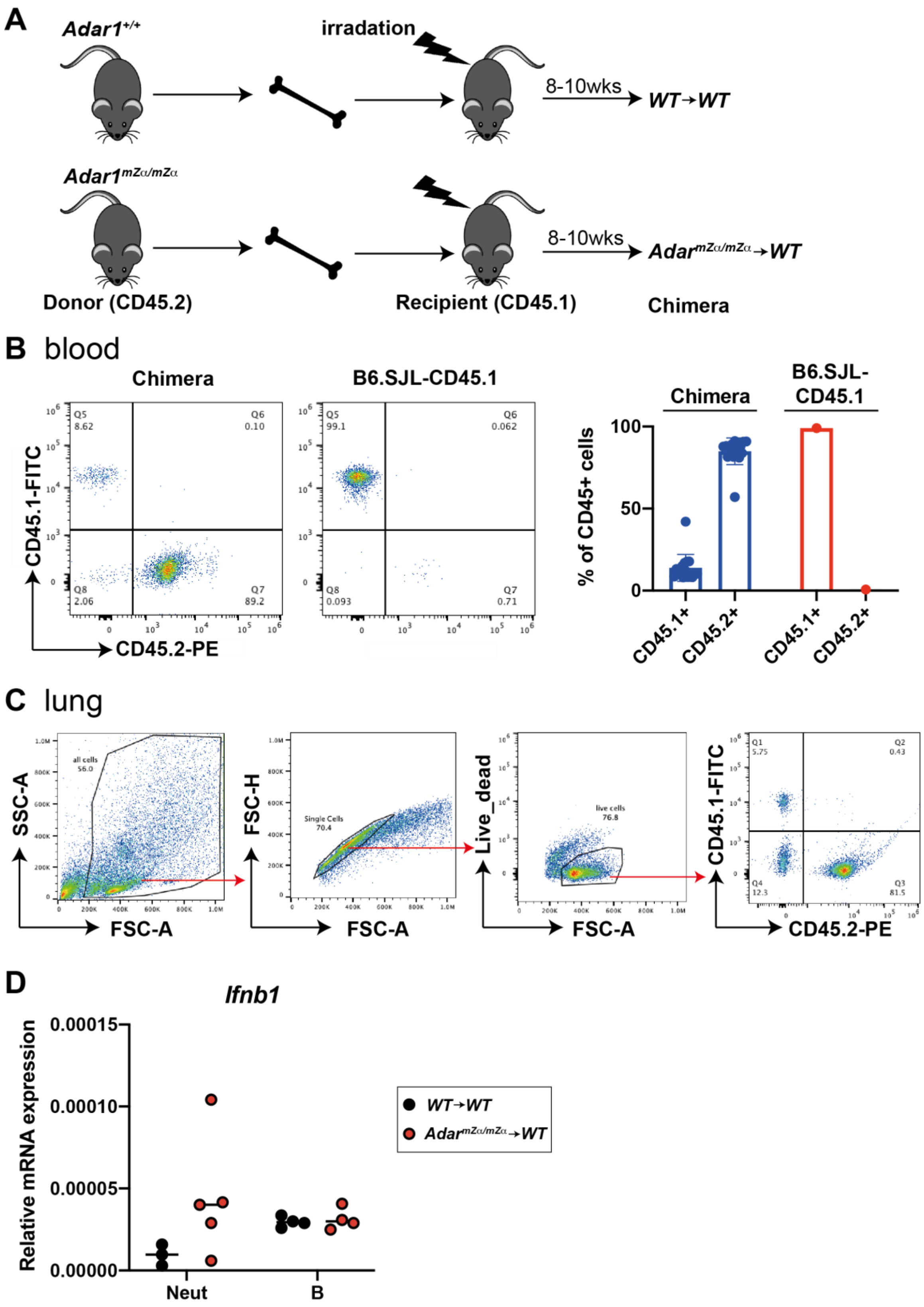
A
Adar1+/+
irradiation
8-10wks
WT→WT
Adar1mZα/mZα
8-10wks
AdarmZα/mZα→WT
Donor (CD45.2)
Recipient (CD45.1)
Chimera
B blood
Chimera
B6.SJL-CD45.1
CD45.1-FITC
CD45.2-PE
Q5 8.62
Q6 0.10
Q8 2.06
Q7 89.2
Q5 99.1
Q6 0.062
Q8 0.093
Q7 0.71
% of CD45+ cells
Chimera
B6.SJL-CD45.1
CD45.1+
CD45.2+
C lung
SSC-A
FSC-A
all cells 56.0
FSC-H
Single Cells 70.4
Live_dead
live cells 76.8
Q1 5.75
Q2 0.43
Q4 12.3
Q3 81.5
D
Ifnb1
Relative mRNA expression
WT→WT
AdarmZα/mZα→WT
Neut
B

Figure S5, related to Figure 4D. Analysis of BM chimeras.

A. Schematic representation of the generation of BM chimeric animals.

B. White blood cells from BM chimeric mice and, as control, from an untreated B6.SJL-CD45.1 animal, were analysed by FACS. Cells were gated on single, live cells. Representative FACS plots from a WT→WT animal (left) and pooled data from two independent experiments involving a total seven WT→WT and eight *Adar1*$^{mZ\alpha/mZ\alpha}$→WT BM chimeric animals (right) are shown. Bars show the mean and error bars represent SD.

C. Lung cells from BM chimeric mice were analysed by FACS. Data from a representative WT→WT animal are shown.

D. Neutrophil and B cell RNA samples obtained from the lungs of BM chimeric animals were tested by conventional RT qPCR for *Ifnb1* transcript levels. mRNA levels are shown relative to *Actb*. Each dot represents samples from an individual mouse.

Figure S6.

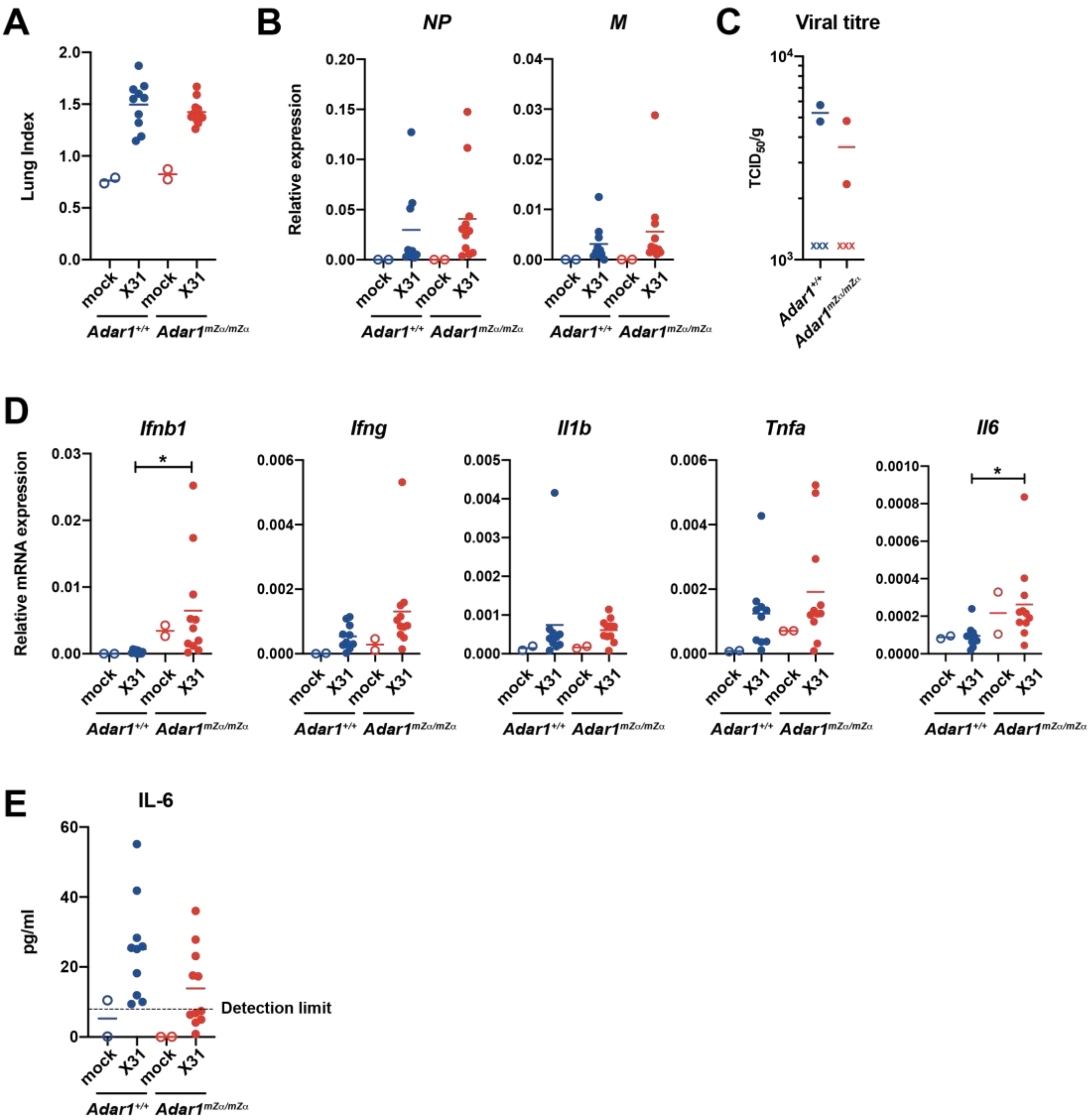

A
B
C
D
E
Lung Index
Relative expression
NP
M
Viral titre
TCID50/g
Relative mRNA expression
Ifnb1
Ifng
Il1b
Tnfa
Il6
IL-6
pg/ml
Detection limit
mock
X31
Adar1+/+
Adar1mZα/mZα

Figure S6. Analysis of WT and *Adar1*$^{mZ\alpha/mZ\alpha}$ mice on day 7 after IAV infection. Related to Figure 5.

A-E. WT or *Adar1*$^{mZ\alpha/mZ\alpha}$ mice were infected intranasally with 0.04 HAU of IAV strain A/X-31. On day 7 post infection, lungs and sera were collected.

A. The 'lung index' was calculated (lung weight/body weight x100).

B. Levels of the viral *NP* and *M* transcripts were analysed by RT-qPCR in RNA samples extracted from total lung. Data are shown relative to *Actb* (*NP*) or *Gapdh* (*M*).

C. Lung viral titres were determined in samples from infected animals by $TCID_{50}$ analysis and were normalised to lung weight. Crosses indicate samples without detectable viral titre.

D. Levels of the indicated mRNAs were determined as in (B).

E. Serum IL-6 concentrations were analysed by ELISA.

In (A-B and D-E), pooled data from two independent experiments (mock infected: n=2 mice per genotype; A/X-31-infected: n=10 WT and n=11 *Adar1*$^{mZ\alpha/mZ\alpha}$ mice) are shown. In (C), data from one experiment with n=5 infected mice per genotype are shown. Each dot represents an individual mouse and the mean is indicated (*$p<0.05$, unpaired t test).

Figure S7.

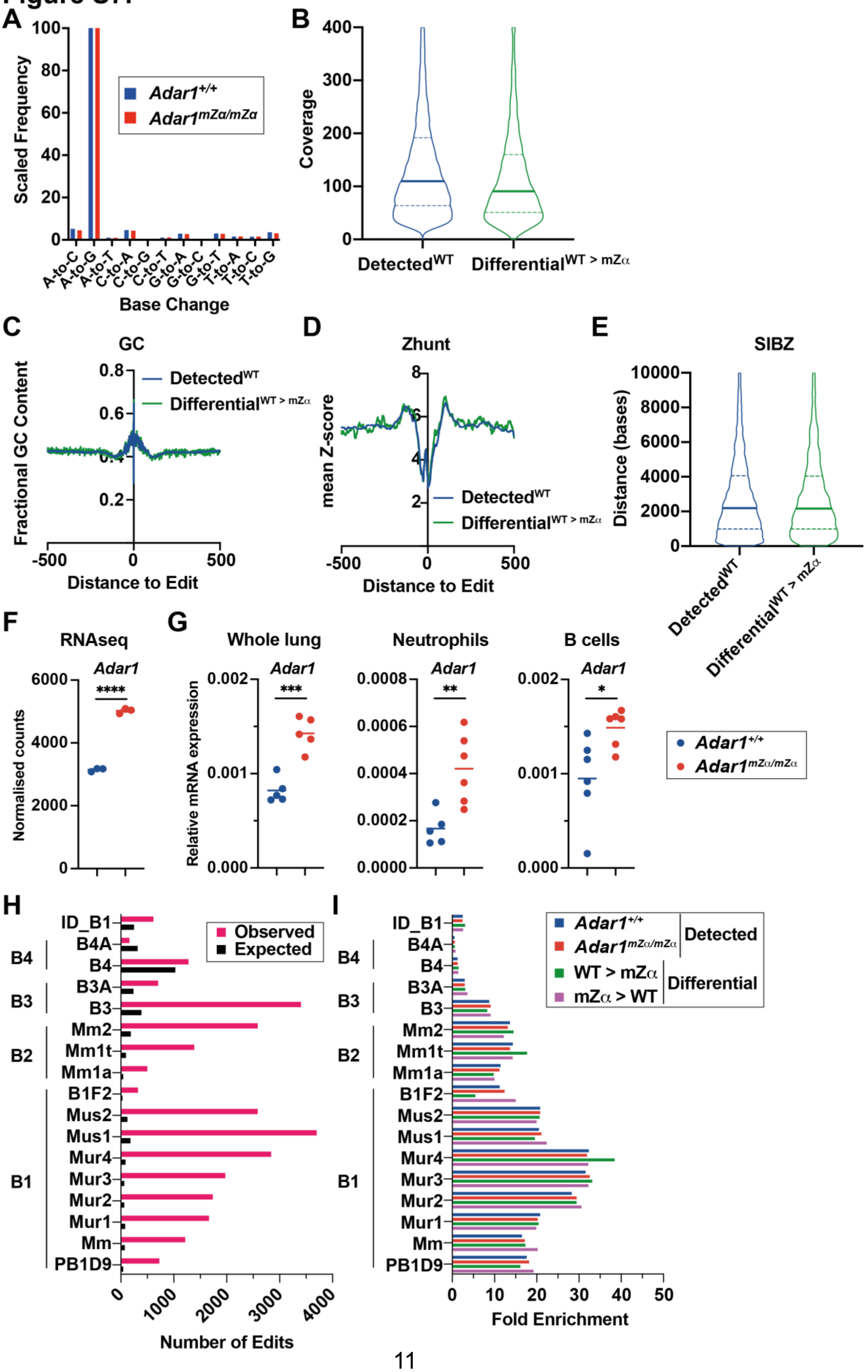

A
Scaled Frequency
Adar1+/+
Adar1mZα/mZα
A-to-C A-to-G A-to-T C-to-A C-to-G C-to-T G-to-A G-to-C G-to-T T-to-A T-to-C T-to-G
Base Change
B
Coverage
DetectedWT
DifferentialWT > mZα
C
GC
Fractional GC Content
Distance to Edit
D
Zhunt
mean Z-score
Distance to Edit
E
SIBZ
Distance (bases)
F
RNAseq
Adar1
Normalised counts
G
Whole lung
Neutrophils
B cells
Relative mRNA expression
H
Observed
Expected
Number of Edits
I
Detected
Differential
WT > mZα
mZα > WT
Fold Enrichment
ID_B1
B4A
B4
B3A
B3
Mm2
Mm1t
Mm1a
B1F2
Mus2
Mus1
Mur4
Mur3
Mur2
Mur1
Mm
PB1D9

Figure S7. Analysis of RNA editing. Related to Figure 7.

A. Differences between the reference genome and RNAseq data were analysed for all possible base changes. The frequency of A-to-G changes was set to 100 for WT and *Adar1*$^{mZ\alpha/mZ\alpha}$ lung samples.

B. Sequence coverage for 'detected^{WT}' and 'differential$^{WT>mZ\alpha}$' sites is shown in violin plots. Solid horizontal lines show the median and dotted lines indicate quartiles.

C. Nucleotide composition was analysed in 1001 nt windows centred on editing sites detected in WT mice or on 'differential$^{WT>mZ\alpha}$' sites. The fractional GC content is shown.

D. The Zhunt algorithm was used to calculate the propensity of sequences within 500 nt from editing sites to form the Z conformation. Log10 transformed mean z-scores are shown for 'detected^{WT}' and 'differential$^{WT>mZ\alpha}$' sites.

E. The distances of editing sites to genomic regions predicted by SIBZ to form the Z-conformation is shown in violin plots. Solid horizontal lines show the median and dotted lines indicate quartiles.

F. Normalised *Adar1* read counts from lung RNA sequencing (see Figure 2) are shown.

G. *Adar1* mRNA levels were analysed by RT-qPCR in RNA samples extracted from whole lungs (left) or from FACS-enriched lung neutrophils (middle) or lung B cells (right) of WT and *Adar1*$^{mZ\alpha/mZ\alpha}$ animals. Data are shown relative to *Actb*.

H. Sub-families of SINEs were analysed as in Figure 7D, but are plotted to include those where observed or expected values exceeded 250.

I. Fold enrichments of editing events were calculated relative to expected numbers of edits for SINE sub-families using editing events detected in WT or *Adar1*$^{mZ\alpha/mZ\alpha}$ samples and differentially edited sites.

In (F) and (G), each dot represents an individual mouse and horizontal lines show the mean. In (F), lungs from 3 mice per genotype were sequenced. In (G),

samples from 5-6 mice per genotype were analysed. Data were analysed by unpaired t test (****$p<0.0001$, ***$p<0.001$, **$p<0.01$, *$p<0.05$).

Table S2. qPCR probes and primers. Related to STAR Methods.

**Taqman Probes**	**Assay Probe ID**
Ifnb1	Mm00439552_s1
Ifng	Mm01168134_m1
Tnfa	Mm00443258_m1
Il1b	Mm00434228_m1
Il6	Mm00446191_m1
Ifit1	Mm00515153_m1
Ifit2	Mm00492606_m1
Ifi44	Mm00505670_m1
Isg15	Mm01705338_s1
Isg20	Mm00469585_m1
Zbp1	Mm01247052_m1
Oas1a	Mm00836412_m1
NP	Custom probe based on NC_002019.1<br>Assay ID:AIX02UC
Actin	Mm02619580_g1
Gapdh	Mm99999915_g1 (4352932E)
**SYBR Green oligos**	**Sequence**
M	F: 5'-CTTCTAACCGAGGTCGAAACGTA<br>R: 5'-GGTGACAGGATTGGTCTTGTCTTTA<br>from: Shin et al. 2013, Virology Journal 10:303
Gapdh	F: 5'-CATGGCCTTCCGTGTTCCTA<br>R: 5'-CCTGCTTCACCACCTTCTTGAT<br>from: Tsujita et al. 2006, PNAS 103:11946
