# Supplementary material for: Adenosine-to-inosine editing of endogenous Z-form RNA by the deaminase ADAR1 prevents spontaneous MAVS-dependent type I interferon responses
Source: Immunity. 2021 Sep 14;54(9):1961–1975.e5. doi: 10.1016/j.immuni.2021.08.011 (PMC8459395; doi:10.1016/j.immuni.2021.08.011)
Supplement: Document S2. Article plus supplemental information [file mmc3.pdf]

Immunity

Article

Adenosine-to-inosine editing of endogenous Z-form RNA by the deaminase ADAR1 prevents spontaneous MAVS-dependent type I interferon responses

Graphical abstract

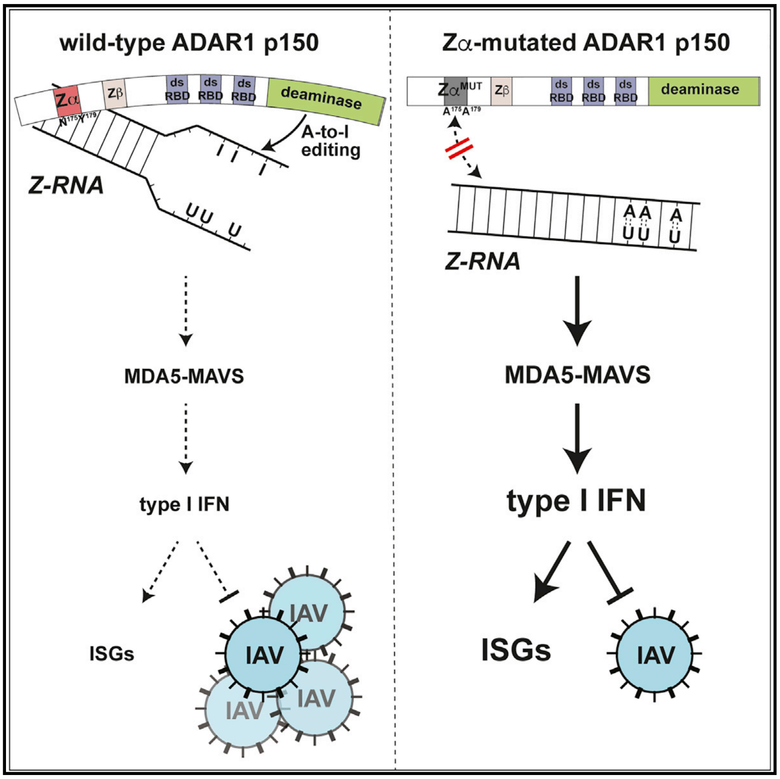

Highlights

- Generation of mice bearing mutations in the ADAR1 Zα domain, disabling Z-RNA binding
- *Adar1*$^{mZ\alpha/mZ\alpha}$ mice express type I IFNs and ISGs in multiple organs, including lung
- The IFN response in *Adar1*$^{mZ\alpha/mZ\alpha}$ mice depends on MAVS and protects against flu
- ADAR1's Zα domain is required for editing of a subset of RNA substrates

Authors

Qiannan Tang, Rachel E. Rigby, George R. Young, ..., Alain R. Townsend, George Kassiotis, Jan Rehwinkel

Correspondence

jan.rehwinkel@imm.ox.ac.uk

In brief

Nucleic acids can adopt the Z-conformation, an unusual left-handed double helix. Tang et al. show that mutations in the Z-RNA recognition domain of the adenosine deaminase ADAR1 result in spontaneous type I interferon production. Analysis of adenosine-to-inosine changes shows enrichment in transposable elements and suggests that editing by ADAR1 curtails the immunostimulatory potential of endogenous Z-form RNAs, with relevance to autoinflammatory disease in humans.

Tang et al., 2021, Immunity *54*, 1961–1975
September 14, 2021 © 2021 The Authors. Published by Elsevier Inc.
https://doi.org/10.1016/j.immuni.2021.08.011

CellPress

Immunity

CellPress
OPEN ACCESS

Article

Adenosine-to-inosine editing of endogenous Z-form RNA by the deaminase ADAR1 prevents spontaneous MAVS-dependent type I interferon responses

Qiannan Tang,[1] Rachel E. Rigby,[1,6] George R. Young,[2,6] Astrid Korning Hvidt,[1] Tanja Davis,[1] Tiong Kit Tan,[1] Anne Bridgeman,[1] Alain R. Townsend,[1,3] George Kassiotis,[4,5] and Jan Rehwinkel[1,7,*]

[1]Medical Research Council Human Immunology Unit, Medical Research Council Weatherall Institute of Molecular Medicine, Radcliffe Department of Medicine, University of Oxford, Oxford, OX3 9DS, UK
[2]Bioinformatics and Biostatistics STP, The Francis Crick Institute, London, NW1 1AT, UK
[3]Centre for Translational Immunology, Chinese Academy of Medical Sciences Oxford Institute, University of Oxford, Oxford, OX3 7FZ, UK
[4]Retroviral Immunology, The Francis Crick Institute, London, NW 1AT, UK
[5]Department of Infectious Disease, Faculty of Medicine, Imperial College London, London, W2 1NY, UK
[6]These authors contributed equally
[7]Lead contact
*Correspondence: jan.rehwinkel@imm.ox.ac.uk
https://doi.org/10.1016/j.immuni.2021.08.011

SUMMARY

Nucleic acids are powerful triggers of innate immunity and can adopt the Z-conformation, an unusual left-handed double helix. Here, we studied the biological function(s) of Z-RNA recognition by the adenosine deaminase ADAR1, mutations in which cause Aicardi-Goutières syndrome. *Adar1*$^{mZ\alpha/mZ\alpha}$ mice, bearing two point mutations in the Z-nucleic acid binding (Zα) domain that abolish Z-RNA binding, displayed spontaneous induction of type I interferons (IFNs) in multiple organs, including in the lung, where both stromal and hematopoietic cells showed IFN-stimulated gene (ISG) induction. Lung neutrophils expressed ISGs induced by the transcription factor IRF3, indicating an initiating role for neutrophils in this IFN response. The IFN response in *Adar1*$^{mZ\alpha/mZ\alpha}$ mice required the adaptor MAVS, implicating cytosolic RNA sensing. Adenosine-to-inosine changes were enriched in transposable elements and revealed a specific requirement of ADAR1's Zα domain in editing of a subset of RNAs. Thus, endogenous RNAs in Z-conformation have immunostimulatory potential curtailed by ADAR1, with relevance to autoinflammatory disease in humans.

INTRODUCTION

The innate immune system monitors the intra- and extracellular environments for unusual nucleic acids (Bartok and Hartmann, 2020). This process, known as "nucleic acid sensing", detects pathogen invasion and disturbances to homeostasis. It involves a large number of germline encoded nucleic acid sensors. Upon engagement by immunostimulatory DNA or RNA, these sensors signal to initiate a large spectrum of responses, including transcription of the genes encoding type I interferons (IFNs). Type I IFNs—secreted cytokines that act in paracrine and autocrine manner—induce expression of hundreds of IFN-stimulated genes (ISGs). The proteins encoded by ISGs mediate a plethora of functions and include antiviral effectors (Schoggins, 2019). Sustained type I IFN responses can have detrimental effects and cause a range of diseases, including the neuroinflammatory Aicardi-Goutières syndrome (AGS) (Uggenti et al., 2019). It is therefore important to understand the molecular mechanisms that prevent activation of nucleic acid sensors by "normal" DNA and RNA present in healthy cells (Bartok and Hartmann, 2020).

We and others proposed that double-stranded (ds) nucleic acids adopting an unusual conformation known as Z-DNA and Z-RNA activate innate immunity (Kesavardhana and Kanneganti, 2020; Maelfait et al., 2017; Sridharan et al., 2017; Zhang et al., 2020b). Z-DNA was initially described by Alexander Rich (Wang et al., 1979). Unlike canonical B-DNA, a right-handed double helix, Z-DNA is a left-handed double helix with a zigzag-shaped phosphodiester back bone (Wang et al., 1979). dsRNA can also adopt the Z-conformation (Davis et al., 1986; Hall et al., 1984). Biological functions of Z nucleic acids, in particular those of Z-RNA, are incompletely understood (Herbert, 2019). A small number of proteins, all involved in innate immunity, contain Z-DNA and Z-RNA binding domains known as Zα domains (Athanasiadis, 2012). These domains specifically bind to and stabilize Z-DNA and Z-RNA or induce the Z-conformation (Athanasiadis, 2012; Brown et al., 2000; Kim et al., 2018; Schwartz et al., 1999).

There are two mammalian proteins with Zα domains: Z-DNA binding protein 1 (ZBP1) and adenosine deaminase acting on RNA 1 (ADAR1; also known as DRADA1). ZBP1 contains two Zα domains that recognize viral and endogenous Z-RNAs (Devos

This is an open access article under the CC BY license (http://creativecommons.org/licenses/by/4.0/).

et al., 2020; Jiao et al., 2020; Maelfait et al., 2017; Sridharan et al., 2017; Wang et al., 2020; Zhang et al., 2020b). Binding to Z-RNA activates ZBP1 and results in the induction of necroptosis, an inflammatory form of cell death (Maelfait et al., 2020).

ADAR1 has two splice isoforms: ADAR1-p110, which is constitutively expressed and localized in the cell nucleus, and ADAR1-p150, which is IFN inducible and present in the nucleus and cytosol. Both isoforms contain a C-terminal deaminase domain that converts adenosine to inosine in dsRNA, a process known as A-to-I RNA editing. Conversion of adenosine to inosine in protein-coding sequences can lead to incorporation of non-synonymous amino acids during translation because of base pairing of inosine with cytosine. However, the vast majority of A-to-I editing events occur in non-coding RNAs (Eisenberg and Levanon, 2018; Reich and Bass, 2019). This includes transcripts from repetitive elements (REs), particularly *Alu* elements and short interspersed nuclear elements (SINEs) in human and mouse, respectively. Both ADAR1 isoforms further contain three dsRNA binding domains (dsRBDs) and a so-called Zβ domain. Zβ adopts a fold similar to Zα domains but does not bind Z-form nucleic acids, because of substitutions of key amino acids (Athanasiadis et al., 2005; Kim et al., 2003). ADAR1-p150 has an extended N terminus harboring a Zα domain (Heraud-Farlow and Walkley, 2020).

ADAR1 deficiency results in inflammatory phenotypes. In human, germline *ADAR1* mutations cause AGS (Rice et al., 2012). These mutations predominantly map to the deaminase domain; however, one compound heterozygous mutation encoding p.Pro193Ala is found in the Zα domain. Pro193 contributes to Z-form nucleic acid binding (Schwartz et al., 1999) and changing it to Ala reduces RNA editing in a reporter assay (Mannion et al., 2014). *Adar1*$^{-/-}$ mice, editing-deficient *Adar1*$^{E861A/E861A}$ animals, and *Adar1*$^{p150-/p150-}$ mice, which only lack ADAR1-p150, all die in utero (Hartner et al., 2004; Liddicoat et al., 2015; Wang et al., 2004; Ward et al., 2011). Akin to spontaneous type I IFN induction in AGS patients with *ADAR1* mutation, *Adar1*$^{-/-}$ and *Adar1*$^{E861A/E861A}$ embryos display type I IFN responses prior to death (Hartner et al., 2009; Liddicoat et al., 2015). Multiple nucleic acid sensors mediate the anti-proliferative, cell death, and type I IFN phenotypes in ADAR1-deficient settings: the oligoadenylate synthetase (OAS)-RNase L system (Li et al., 2017), protein kinase R (PKR) (Chung et al., 2018; Li et al., 2010), and melanoma differentiation-associated protein 5 (MDA5) (Liddicoat et al., 2015; Mannion et al., 2014; Pestal et al., 2015). Upon activation by dsRNA, OAS proteins synthesize 2′-5′ oligoadenylate, a second messenger that in turn activates RNase L, resulting in widespread RNA degradation. PKR also detects dsRNA and represses protein translation. Both effects may explain the lethality of ADAR1-deficient cells (Chung et al., 2018; Li et al., 2017). Induction of type I IFNs in ADAR1-deficient mice and human cells is mediated by the RNA sensor MDA5, which signals via its adaptor mitochondrial antiviral-signaling protein (MAVS) (Bajad et al., 2020; Chung et al., 2018; Guallar et al., 2020; Liddicoat et al., 2015; Mannion et al., 2014; Pestal et al., 2015).

These observations suggest a model in which endogenous dsRNAs are stabilized in ADAR1-deficient cells because of the absence of RNA editing and are then recognized by RNA sensors (Dias et al., 2019; Eisenberg and Levanon, 2018). Some ADAR1 substrates such as transcripts from *Alu* elements base pair to form duplex structures, which may be destabilized by inosine: uracil mismatches introduced by RNA editing (Ahmad et al., 2018; Chung et al., 2018; Pfaller et al., 2018; Song et al., 2020). In an RNase protection assay, transcripts spanning *Alu* elements in inverted orientation are protected by recombinant MDA5 protein in RNA samples extracted from ADAR1-deficient cells (Ahmad et al., 2018; Mehdipour et al., 2020). However, how the different nucleic acid binding domains in ADAR1 select and recruit RNA substrates for subsequent editing is unknown.

We hypothesized that Z-form nucleic acid binding by the Zα domain in ADAR1-p150 regulates innate immunity. Here, we generated mice bearing two missense mutations in the Zα domain, which prevent nucleic acid binding. Although these *Adar1*$^{mZ\alpha/mZ\alpha}$ mice were developmentally normal and fertile, they displayed spontaneous induction of type I IFNs and ISGs in multiple organs and cell types, including neutrophils in the lung. This phenotype conferred partial protection against influenza A virus (IAV) infection and was dependent on MAVS. Analysis of sequencing data revealed that ~8% of RNA editing events in wild-type (WT) cells required a functional ADAR1-p150 Zα domain. Taken together, our findings suggest that recognition of Z-form RNA by ADAR1 contributes to the suppression of IFN responses.

RESULTS

Generation of Zα domain mutated mice

To study the role of Z-form nucleic acid binding to the Zα domain in ADAR1-p150 in an *in vivo* setting, we generated mice bearing two missense mutations: p.Asn175Ala and p.Tyr179Ala. These residues are conserved and are homologous to Asn173 and Tyr177 in human ADAR1; they were chosen because of their essential role in Z-form nucleic acid binding (Feng et al., 2011; Li et al., 2009; Schade et al., 1999; Schwartz et al., 1999). Given the embryonic lethality of *Adar1*$^{p150-/p150-}$ mice (Ward et al., 2011), we opted for a conditional strategy (Figure S1A). The Zα domain is encoded by exon 2 of the *Adar1* gene. In brief, we introduced in inverted orientation into the intron between exons 2 and 3 a mutated copy of exon 2 (designated 2*) containing four nucleotide substitutions, changing both Asn175 and Tyr179 to Ala. We flanked exons 2 and 2* with LoxP and Lox2272 sites such that Cre-mediated recombination removes exon 2 and flips exon 2* into forward orientation (Figure S1A). We designated the conditional allele "*fl-mZα*" and the recombined allele expressing mutant Adar1 "*mZα*." To determine the impact of the Zα domain mutations when present in all cells and tissues, we crossed *Adar1*$^{+/fl\text{-}mZ\alpha}$ mice with a line expressing Cre recombinase under control of the ubiquitously active *Pgk* promoter. The resulting *Adar1*$^{+/mZ\alpha}$ mice were intercrossed to generate homozygous animals. We validated the presence of the mutations by sequencing and found that *Adar1*$^{mZ\alpha/mZ\alpha}$ animals were born at expected mendelian ratios (Figures S1B and S1C). Furthermore, they developed normally and were fertile. The mutations introduced into the Zα domain did not alter the expression of the two isoforms of ADAR1 in bone marrow-derived myeloid cells (BMMCs) (Figure S1D). Taken together, Z-form nucleic acid binding by ADAR1-p150 was not essential for survival at whole organism level.

CellPress
OPEN ACCESS

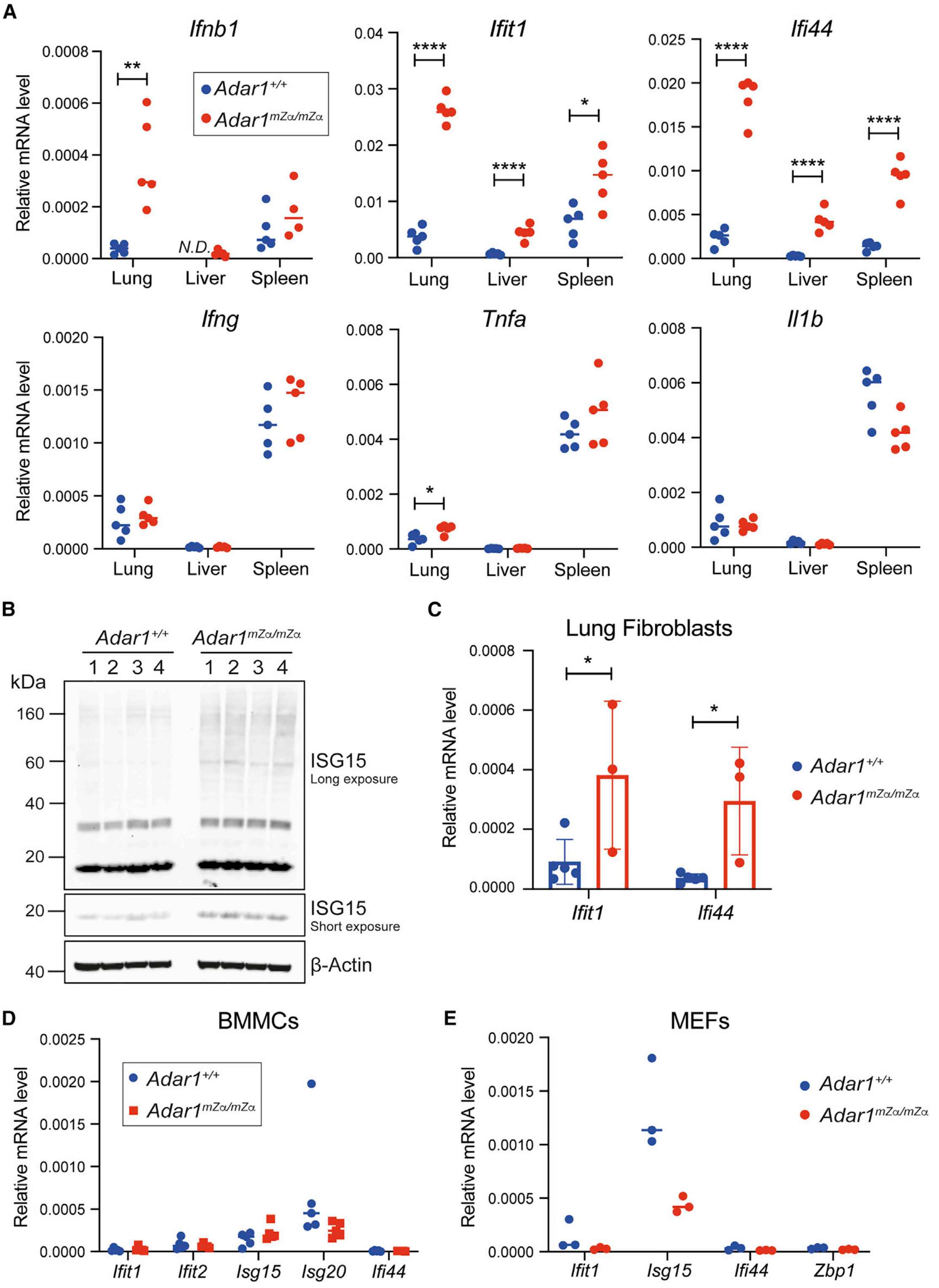

Figure 1. Mutation of ADAR1-p150's Zα domain triggers spontaneous type I IFN responses in multiple organs

(A) Levels of the indicated mRNAs were analyzed using qRT-PCR in RNA samples extracted from tissues of WT and *Adar1*$^{mZ\alpha/mZ\alpha}$ animals and are shown relative to *Gapdh*. Each dot represents an individual mouse. N.D., not detectable.

(B) Protein extracts from whole lungs from animals of the indicated genotypes were used for western blot with an α-ISG15 antibody. β-Actin served as a loading control. Each lane represents a sample from an individual mouse.

(legend continued on next page)

Mutation of the Zα domain in ADAR1-p150 triggers a multi-organ type I IFN response

We tested whether *Adar1*$^{mZ\alpha/mZ\alpha}$ mice display spontaneous activation of type I IFNs as was reported in ADAR1-deficient settings. We collected lung, liver, and spleen tissues and extracted RNA for qRT-PCR analysis. Transcript levels of *Ifnb1* (encoding IFNβ) were elevated in lung RNA samples from *Adar1*$^{mZ\alpha/mZ\alpha}$ mice (Figure 1A). Moreover, the ISG transcripts *Ifit1* and *Ifi44* were expressed at higher levels in all three organs from Zα domain mutated animals (Figure 1A). This gene expression signature was type I IFN specific: tissues from *Adar1*$^{mZ\alpha/mZ\alpha}$ mice contained comparable mRNA levels of *Ifng*, *Tnfa*, and *Il1b* (encoding IFNγ, TNF-α, and pro-IL-1β), with only a minor increase of *Tnfa* mRNA in lung (Figure 1A). We validated the ISG signature at protein level by analyzing expression of ISG15 in whole lung lysates. ISG15 is a ubiquitin-like modifier that is induced by IFN and is conjugated to target proteins in a process called ISGylation (Perng and Lenschow, 2018). Western blot showed that lung lysates from *Adar1*$^{mZ\alpha/mZ\alpha}$ mice contained increased amounts of monomeric ISG15 as well as increased levels of ISGylated proteins, visible as a high-molecular weight smear (Figure 1B).

We next analyzed different types of cultured primary cells from WT and *Adar1*$^{mZ\alpha/mZ\alpha}$ mice. We observed heightened ISG expression in Zα domain mutated lung fibroblasts (Figure 1C). In contrast, comparable levels of ISG transcripts were found in WT and *Adar1*$^{mZ\alpha/mZ\alpha}$ BMMCs, as well as in mouse embryonic fibroblasts (MEFs) (Figures 1D and 1E). This indicated that ISG induction was cell type specific.

Among the organs we analyzed, lung exhibited the most profound ISG signature, and spontaneous ISG induction was also observed in cultured lung fibroblasts (Figures 1A and 1C). We therefore focused on the lung for the next set of experiments. We used RNA extracted from lung for RNA sequencing (RNA-seq) to obtain a global view of gene expression in *Adar1*$^{mZ\alpha/mZ\alpha}$ mice. We found that 99 protein coding genes were differentially expressed by at least 2-fold in mutant lungs, including 89 transcripts with increased expression and 10 transcripts with decreased expression (Figure 2A). Forty percent of the induced mRNAs were encoded by ISGs; this included well-known factors such as *Irf7*, *Cxcl10*, *Zbp1*, and *Usp18* (Figure 2A). We used Gene Ontology (GO) analysis of biological processes to further classify genes with increased expression. GO terms related to type I IFNs and antiviral defense were enriched among genes induced in *Adar1*$^{mZ\alpha/mZ\alpha}$ lungs (Figure 2B). The most highly enriched GO category, regulation of ribonuclease activity, included many *Oas* transcripts, which are IFN inducible (Schoggins, 2019).

In addition to the activation of MDA5, loss of ADAR1 also results in activation of the OAS-RNase L system and PKR. The latter may be particularly important in cancer settings (Gannon et al., 2018; Ishizuka et al., 2019; Liu et al., 2019). Although PKR activation results in a global shutdown of translation, some proteins are selectively made and mediate the integrated stress response (ISR) (Pakos-Zebrucka et al., 2016). These include the transcription factor ATF4, which induces ISR genes. Interestingly, a recent study reported induction of ISR genes in heterozygous *Adar1*$^{P195A/p150-}$ mice (Maurano et al., 2021). We observed induction of some ATF4-dependent genes (Harding et al., 2003) in *Adar1*$^{mZ\alpha/mZ\alpha}$ mice, including *Asns* (1.3-fold), *Slc7a5* (1.3-fold), *Slc7a11* (1.9-fold), and *Mthfd2* (1.7-fold) (Figure S1E). It is therefore possible that the Zα domain was required to limit not only type I IFN induction but also PKR-dependent stress responses.

We also analyzed REs in our RNA-seq data and found 540 induced and 112 repressed REs (Figure 2C; Table S1). SINEs were underrepresented among differentially expressed REs, while long terminal repeat (LTR) elements were enriched (Figure 2C). Dysregulation of REs occurs in settings of inflammation and can be driven by IFNs (Chuong et al., 2016). Taken together, these data showed that the lungs of Zα domain mutated animals displayed a type I IFN-driven gene signature.

Stromal and hematopoietic cells contribute to the ISG signature in the lungs of *Adar1*$^{mZ\alpha/mZ\alpha}$ mice

To identify the type of cell(s) that display the ISG signature in the lungs of *Adar1*$^{mZ\alpha/mZ\alpha}$ mice, we used magnetic-activated cell sorting (MACS) to isolate hematopoietic cells, marked by CD45 expression, and stromal cells lacking this marker. There was no difference in the proportions of hematopoietic and stromal cells between WT and *Adar1*$^{mZ\alpha/mZ\alpha}$ lungs (Figure 3A). We confirmed the purity of MACS-enriched hematopoietic and stromal cells to be >97% (Figure S2). We then extracted RNA for qRT-PCR analysis of ISGs and found that both lung hematopoietic and stromal cells displayed ISG induction (Figure 3B). For the three ISGs tested, fold inductions in *Adar1*$^{mZ\alpha/mZ\alpha}$ samples were similar in CD45+ and CD45− cells (Figure 3B). However, it is noteworthy that baseline ISG levels were lower in hematopoietic cells compared with stromal cells (Figure 3B).

Next, we analyzed ISG expression in different types of lung hematopoietic and stromal cells. Using two staining panels and fluorescence-activated cell sorting (FACS), we obtained eight different populations of CD45+ cells, including B cells, T cells, dendritic cells (DCs), monocytes, macrophages, natural killer (NK) cells, neutrophils, and eosinophils (Figures S3A and S3B). There were no differences between WT and *Adar1*$^{mZ\alpha/mZ\alpha}$ mice in the proportions of these cell populations, with the exception of DCs, which were less abundant in the mutant lungs (Figure S3A). *Ifit1*, *Ifit2*, and *Isg15* were induced in neutrophils from the lungs of *Adar1*$^{mZ\alpha/mZ\alpha}$ mice (Figures 4A and 4B). However, these ISGs showed no, or limited and statistically nonsignificant, expression changes in other types of CD45+ cells (Figures 4A and 4B). In contrast, the ISGs *Ifi44* and *Oas1a* were expressed at increased levels in multiple hematopoietic cell types (Figure 4A). Transcripts encoding type I IFNs were undetectable by qRT-PCR in most samples analyzed, with Ct values similar to those in control reactions without reverse transcriptase. We therefore adapted our qRT-PCR protocol and performed reverse

(C–E) mRNA levels of the indicated ISGs were analyzed using qRT-PCR from cultured lung fibroblasts (C), BMMCs (D), and MEFs (E) of the indicated genotypes and are shown relative to *Gapdh*. Each dot represents cells derived from an individual mouse.

Pooled data from biological replicates are shown with mean (A, D, and E) or mean ± SD (C) and were analyzed using unpaired t test (*p < 0.05, **p < 0.01, and ****p < 0.0001). See also Figure S1.

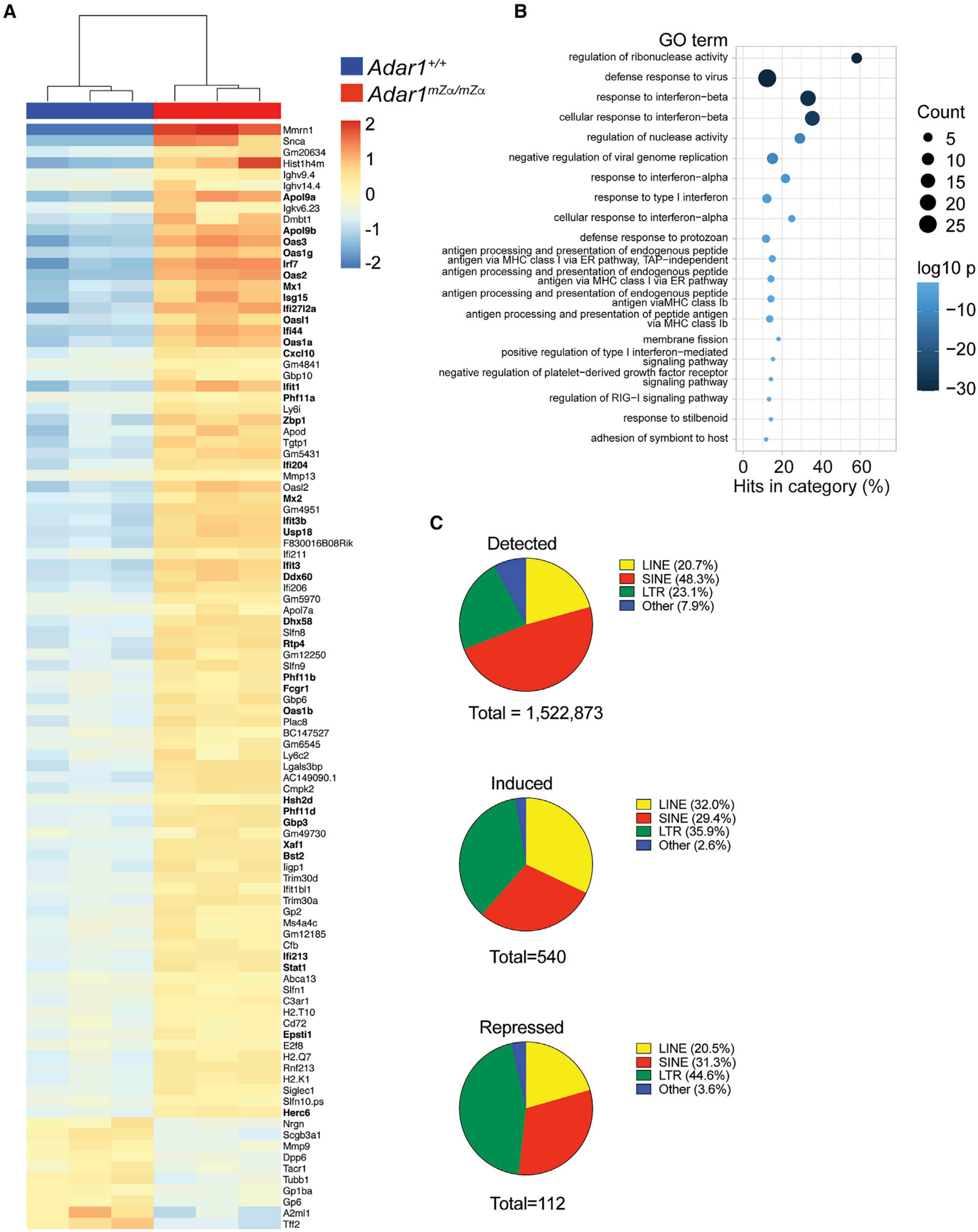

Figure 2. ***Adar1$^{mZ\alpha/mZ\alpha}$* lungs display a type I IFN gene signature**

Total RNA was extracted from lungs of three WT and three *Adar1$^{mZ\alpha/mZ\alpha}$* mice. Ribosomal RNAs were depleted before random-primed library preparation and RNA sequencing. About 100 million reads were obtained per sample.

(A) Differentially expressed genes were defined as displaying a fold change of ≥2 with an adjusted p value < 0.01. The 89 induced and 10 repressed genes were ordered by decreasing fold change and the data were clustered by sample. ISGs are indicated in bold.

(legend continued on next page)

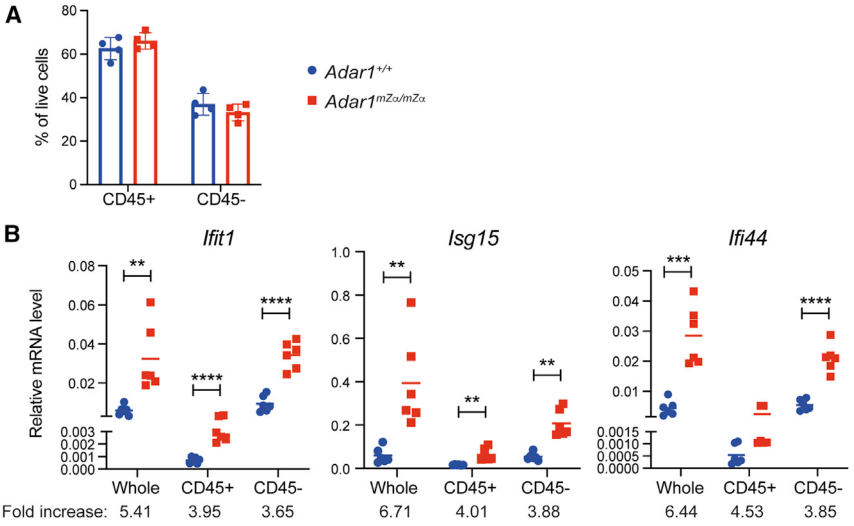

Figure 3. Stromal and hematopoietic cells induce ISGs in *Adar1*$^{mZ\alpha/mZ\alpha}$ lungs

(A) The proportion of hematopoietic (CD45+) and stromal (CD45−) cells in WT and *Adar1*$^{mZ\alpha/mZ\alpha}$ lungs is shown.

(B) mRNA levels of the indicated ISGs were analyzed by qRT-PCR using RNA extracted from whole lung, or from CD45+ or CD45− cells, and are shown relative to *Actb*. Fold increases relative to WT samples were calculated.

Data points represent individual animals. In (A), data from a representative experiment are shown with mean ± SD. In (B), pooled data from two independent experiments including a total of six animals per genotype are shown with mean and were analyzed using unpaired t test (**p < 0.01, ***p < 0.001, and ****p < 0.0001). See also Figure S2.

transcription (RT) with gene-specific primers. As a control, we included primers for RT of *Ifit1* and *Ifi44*. The qPCR data obtained using these cDNAs matched those from conventional qRT-PCR: *Ifit1* mRNA was induced more strongly in neutrophils compared with B cells (Figure S3C). Vice versa, *Ifi44* was induced in B cells but not in neutrophils. We were unable to detect transcripts encoding IFNβ and three selected IFNα subtypes with this target-specific RT method. As a control, total RNA extracted from the lungs of WT mice infected with IAV PR8 or a mutant PR8 virus known to induce higher levels of type I IFNs (Rigby et al., 2019) contained measurable *Ifnb1* transcript levels, validating the gene-specific RT approach (Figure S3D).

Several ISGs, including *Ifit1*, *Ifit2*, and *Isg15*, are not only induced by IFNAR signaling but can also be induced by IRF3 and IRF7, independent of type I IFN-initiated JAK-STAT activation (Ashley et al., 2019; DeFilippis et al., 2006; Goubau et al., 2009; Grandvaux et al., 2002; Lazear et al., 2013). Induction of this ISG subset can therefore occur in a cell-autonomous manner, for example, when aberrant nucleic acids are sensed by pattern recognition receptors (PRRs) that signal via IRF3 and IRF7. Other ISGs, including *Ifi44* and *Oas1a*, are induced only via IFNAR signaling (Lazear et al., 2013). Therefore, our observation that *Ifit1*, *Ifit2*, and *Isg15* were induced primarily in neutrophils, while *Ifi44* and *Oas1a* were induced more broadly (Figures 4A and 4B), suggested that *Adar1*$^{mZ\alpha/mZ\alpha}$ neutrophils autonomously activated IRF3. We further speculate that the ISG signature in other hematopoietic cells such as B cells was due to paracrine type I IFN signaling via IFNAR.

We also analyzed the lung stromal compartment and isolated by FACS endothelial and epithelial cells using CD31 and CD326 as markers, respectively (Figure S4). We further isolated CD45− cells lacking these markers; this mixed population likely included fibroblasts and other cell types. We observed induction of *Ifit1*, *Isg15*, and *Ifi44* in *Adar1*$^{mZ\alpha/mZ\alpha}$ cells of all three populations analyzed (Figure 4C). It is therefore likely that in addition to neutrophils, multiple non-hematopoietic cells initiated type I IFN production in the lungs of *Adar1*$^{mZ\alpha/mZ\alpha}$ mice.

Hematopoietic cells are sufficient to induce ISGs in *Adar1*$^{mZ\alpha/mZ\alpha}$ mice

To further dissect the cellular requirements for ISG induction in *Adar1*$^{mZ\alpha/mZ\alpha}$ mice, we generated bone marrow (BM) chimeric animals. Lethally irradiated *Cd45.1* WT recipients were reconstituted with *Cd45.2* BM from WT or *Adar1*$^{mZ\alpha/mZ\alpha}$ animals (Figure S5A). We found reconstitution levels of the hematopoietic compartment to be about 90% by analyzing cell surface levels of CD45.1 and CD45.2 on white blood cells from recipient mice (Figure S5B). Similar levels of reconstitution were observed in the lung (Figure S5C). We first obtained RNA from whole-lung tissue for qRT-PCR analysis. The ISGs *Ifit1*, *Oas1a*, and *Zbp1* were induced in *Adar1*$^{mZ\alpha/mZ\alpha}$ → WT chimeras compared with WT → WT chimeras (Figure 4D, "Total"). This indicated that *Adar1*$^{mZ\alpha/mZ\alpha}$ BM-derived cells triggered an IFN response in WT animals. We also isolated different cell populations from the lungs of chimeric animals using FACS (see Figure S3 for gating), extracted RNA, and performed qRT-PCR. *Ifit1* and *Ifit2* mRNA levels were induced in neutrophils from *Adar1*$^{mZ\alpha/mZ\alpha}$ → WT chimeras, but not in B cells and CD45− cells (Figure 4D). In contrast, *Oas1a* and *Zbp1* were induced in multiple cell populations, including CD45− cells, neutrophils, and B cells (Figure 4D). These observations were consistent with the results shown in Figures 4A and 4B and indicated that neutrophils initiated type I IFN production that then resulted in ISG induction in other cell types. Consistent with this model, we observed a trend, albeit statistically nonsignificant, toward increased *Ifnb1* mRNA levels in *Adar1*$^{mZ\alpha/mZ\alpha}$ neutrophils, but not in B cells (Figure S5D). Taken together, *Adar1*$^{mZ\alpha/mZ\alpha}$

(B) GO analysis of induced genes. The top 20 GO terms (biological processes), ranked and ordered by p value, are shown. Diameters indicate the number of induced genes assigned to the GO term and colors show the p value.

(C) Detected and differentially expressed REs were assigned to the indicated classes and are shown as pie charts. Differentially expressed REs were identified as having a minimum fold change of 2 and an adjusted p value of less than 0.01.

See also Figure S1 and Table S1.

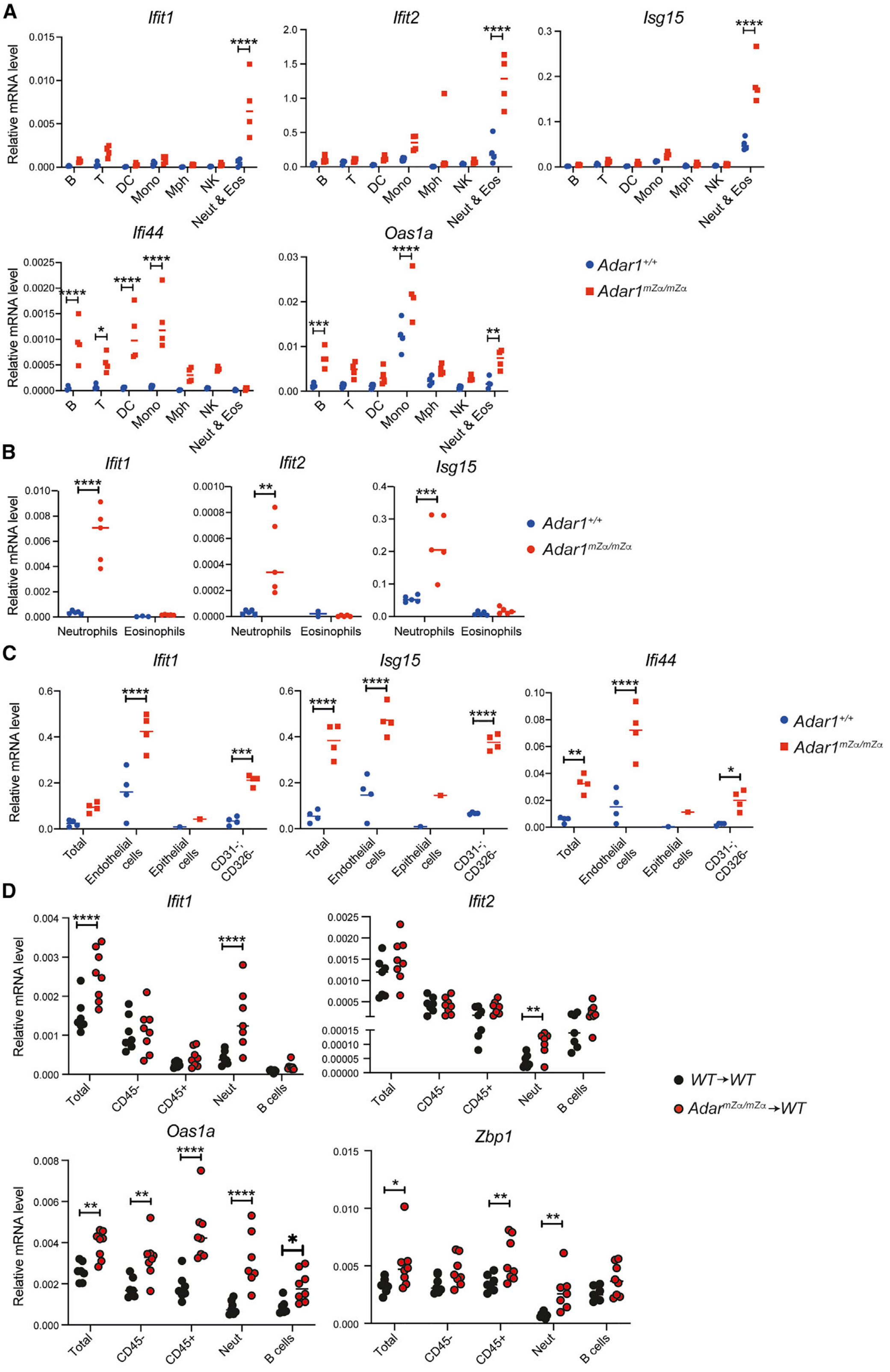

(legend on next page)

hematopoietic cells were sufficient to induce ISGs in the lungs of WT mice.

IAV replication is inhibited in *Adar1*$^{mZ\alpha/mZ\alpha}$ mice

In light of the spontaneous type I IFN response in *Adar1*$^{mZ\alpha/mZ\alpha}$ mice, we next asked whether these Zα domain mutant mice were protected against viral infection. ISGs induced in the lungs of *Adar1*$^{mZ\alpha/mZ\alpha}$ mice included factors such as *Ifit1* and *Zbp1* that control IAV (Pichlmair et al., 2011; Zhang et al., 2020b) (Figure 2A). We intranasally infected WT and *Adar1*$^{mZ\alpha/mZ\alpha}$ mice with an H3N2 strain of recombinant IAV (A/X-31; A/HongKong/1/1968) using a previously determined sublethal dose that causes <20% weight loss in C57BL/6 mice. WT animals lost about 10% body weight from day 3 until day 7 after infection and subsequently recovered their weight (Figure 5A). In contrast, *Adar1*$^{mZ\alpha/mZ\alpha}$ mice gained weight until day 4 after infection (Figure 5A). This was followed by weight loss on days 5–7, which slightly exceeded weight loss in WT mice, and recovery from day 8 onward. These observations suggested that *Adar1*$^{mZ\alpha/mZ\alpha}$ mice were protected against IAV at an early stage of the infection.

We further characterized early and late IAV infection in *Adar1*$^{mZ\alpha/mZ\alpha}$ mice at day 3 and day 7 after inoculation. We calculated a "lung index" (lung weight/body weight × 100; Luo et al., 2012) and found this marker of pathology to be higher in infected WT mice compared with *Adar1*$^{mZ\alpha/mZ\alpha}$ animals on day 3 (Figure 5B). Next, we analyzed viral loads by determining the levels of the viral *NP* and *M* transcripts using qRT-PCR. Compared with infected WT lungs, levels of these viral RNAs were reduced in infected *Adar1*$^{mZ\alpha/mZ\alpha}$ lungs on day 3 (Figure 5C). We confirmed this result by determining virus levels by $TCID_{50}$ (median tissue culture infectious dose) analysis and found reduced viral titers in the lungs of *Adar1*$^{mZ\alpha/mZ\alpha}$ mice on day 3 after infection (Figure 5D). Concomitantly, mRNA levels of *Ifnb1*, *Ifng*, *Il1b*, *Tnfa*, and *Il6*, as well as IL-6 protein, were induced in infected WT lungs, and these effects were curtailed in *Adar1*$^{mZ\alpha/mZ\alpha}$ lungs (Figures 5E and 5F). On day 7 after infection, virus levels were reduced compared with day 3, indicating that weight loss and pathology at later stages of the infection were driven by the host response (Figures 5A and S6A–S6C). Inflammatory responses in WT and *Adar1*$^{mZ\alpha/mZ\alpha}$ mice were similar on day 7 (Figures S6D and S6E), although *Ifnb1* mRNA levels were higher in the mutant animals, potentially explaining their slightly increased weight loss at this time point. In sum, IAV replication and virus-induced inflammation were reduced in *Adar1*$^{mZ\alpha/mZ\alpha}$ animals during the early stage of the infection.

ISG induction in *Adar1*$^{mZ\alpha/mZ\alpha}$ mice is MAVS dependent

We next investigated which nucleic acid sensing pathway triggered spontaneous ISG expression in *Adar1*$^{mZ\alpha/mZ\alpha}$ mice. As ADAR1 deficiency results in activation of the MDA5-MAVS pathway (Liddicoat et al., 2015; Mannion et al., 2014; Pestal et al., 2015), we hypothesized that the ISG signature in *Adar1*$^{mZ\alpha/mZ\alpha}$ mice was driven by MAVS. To test this, we crossed *Adar1* mutant mice with *Mavs*$^{-/-}$ animals to generate *Adar1*$^{mZ\alpha/mZ\alpha}$; *Mavs*$^{-/-}$ mice. Loss of MAVS prevented the ISG induction observed in *Adar1*$^{mZ\alpha/mZ\alpha}$ lungs, livers, and spleens (Figure 6A). Because of the neuropathology observed in patients with AGS, we also analyzed brain tissue. *Adar1*$^{mZ\alpha/mZ\alpha}$ mice showed elevated *Ifnb1* expression and an ISG signature in the brain (Figure 6B). Akin to the situation in other tissues, these effects were MAVS dependent (Figure 6B). Moreover, protection of *Adar1*$^{mZ\alpha/mZ\alpha}$ mice against weight loss during the early stages of IAV infection required MAVS (Figure 6C). Taken together, the Zα domain of ADAR1-p150 was involved in preventing MAVS-mediated IFN induction, implicating a role in limiting activation of MDA5 or RIG-I.

The Zα domain of ADAR1-p150 is required for editing of a subset of RNAs

Human ADAR1-p150 bearing the p.Pro193Ala mutation shows reduced editing activity in a reporter assay (Mannion et al., 2014). To identify natural RNA substrates edited by ADAR1-p150 in a Zα domain-dependent manner, we analyzed our RNA-seq data from WT and *Adar1*$^{mZ\alpha/mZ\alpha}$ lungs for A → G transitions. These are indicative of A-to-I RNA editing, as inosine pairs with cytosine during RT. The most sensitive and specific method for annotating this mutational profile compares the fit of Dirichlet models of observed base frequencies between test and control samples (Piechotta et al., 2017). We extended this methodology to allow comparisons of biological replicates, to incorporate the orientation of the originating RNA (revealed by our stranded RNA-seq protocol), to include fine-grained filtering of potential editing sites, and to add a "detection" step, whereby observed base frequencies at potential editing sites are additionally compared with a model of the per-base error rate obtained *de novo* from the dataset. A → G transitions were more frequent than any other possible base change, indicating that we detected RNA editing events and not sequencing errors (Figure S7A).

Considered individually, for each of the three WT and three *Adar1*$^{mZ\alpha/mZ\alpha}$ lung samples analyzed, we detected ~35,000–40,000 editing sites (Figure 7A). Considered as biological replicates, increasing statistical robustness, 40,342 and 46,164 sites were identified that were shared among all three WT or *Adar1*$^{mZ\alpha/mZ\alpha}$ lung samples, respectively. These "detected" sites had a median editing level of ~10% in both WT and *Adar1*$^{mZ\alpha/mZ\alpha}$ samples (Figure 7B). This showed that there was no global defect in RNA editing in *Adar1*$^{mZ\alpha/mZ\alpha}$ mice.

Figure 4. Multiple hematopoietic and non-hematopoietic cell types display ISGs upregulation in *Adar1*$^{mZ\alpha/mZ\alpha}$ lungs

(A–C) mRNA levels of the indicated ISGs were analyzed by qRT-PCR using RNA extracted from cell populations sorted from lungs of WT and *Adar1*$^{mZ\alpha/mZ\alpha}$ mice and are shown relative to *Actb*. B, B cells; T, T cells; DC, dendritic cells; Mono, monocytes; Mph, macrophages; NK, natural killer cells; Neut, neutrophils; Eos, eosinophils; Total, whole lung.

(D) ISG mRNA levels were analyzed as in (A)–(C) in cell populations sorted from lungs of BM chimeric mice and are shown relative to *Gapdh*.

Each data point represents an individual mouse. Because of the small number of epithelial cells recovered, samples were pooled from multiple mice before RNA extraction (C). Pooled data from two (A and B) or three (D) independent experiments are shown with mean (*p < 0.05, **p < 0.01, ***p < 0.001, and ****p < 0.0001, unpaired t test). See also Figures S3–S5.

CellPress
OPEN ACCESS

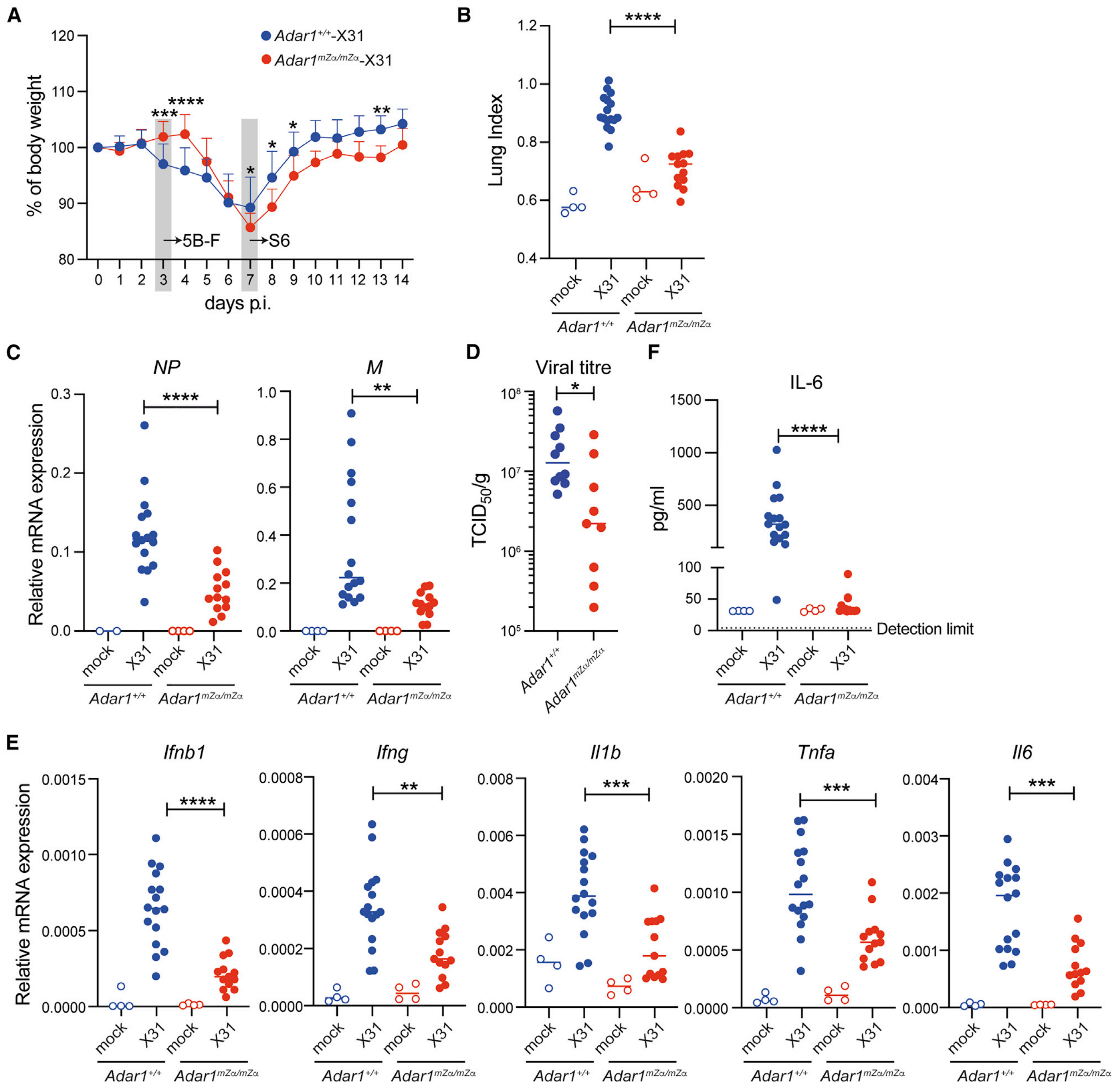

Figure 5. $Adar1^{mZ\alpha/mZ\alpha}$ mice are protected from early IAV infection

(A) WT or $Adar1^{mZ\alpha/mZ\alpha}$ mice were infected intranasally with 0.04 HAU of IAV strain A/X-31. Body weight was monitored daily and is shown as a percentage of starting body weight.

(B–F) WT or $Adar1^{mZ\alpha/mZ\alpha}$ mice were infected as in (A) or mock-infected using viral growth medium. On day 3 post-infection, lungs and sera were collected. (B) A "lung index" was calculated (lung weight/body weight × 100). (C) Levels of the viral *NP* and *M* transcripts were analyzed using qRT-PCR in RNA samples extracted from total lung. Data are shown relative to *Actb* (*NP*) or *Gapdh* (*M*). (D) Lung viral titers were determined in samples from infected animals by $TCID_{50}$ analysis and were normalized to lung weight. (E) Levels of the indicated mRNAs were determined as in (C). (F) Serum IL-6 concentrations were analyzed using ELISA.

In (A), data from three independent experiments including a total of 15 mice per genotype were pooled (mean + SD; *p < 0.05, **p < 0.01, ***p < 0.001, and ****p < 0.0001, mixed-effects analysis). In (B)–(F), pooled data from two independent experiments (mock infected, n = 4 mice per genotype; A/X-31-infected, n = 10–16 WT and n = 9–13 $Adar1^{mZ\alpha/mZ\alpha}$ mice) are shown. Each dot represents an individual mouse and the mean is indicated (**p < 0.01, ***p < 0.001, and ****p < 0.0001, unpaired t test). See also Figure S6.

We further defined "differential$^{WT > mZ\alpha}$" sites as those that were "detected" in the WT samples and showed higher (>2-fold) levels of editing than in $Adar1^{mZ\alpha/mZ\alpha}$ mice. A total of 3,249 sites (8% of sites "detected" in WT) were "differential$^{WT > mZ\alpha}$" and displayed ~9% median editing in WT lungs, which was reduced for the majority of sites in $Adar1^{mZ\alpha/mZ\alpha}$ mice

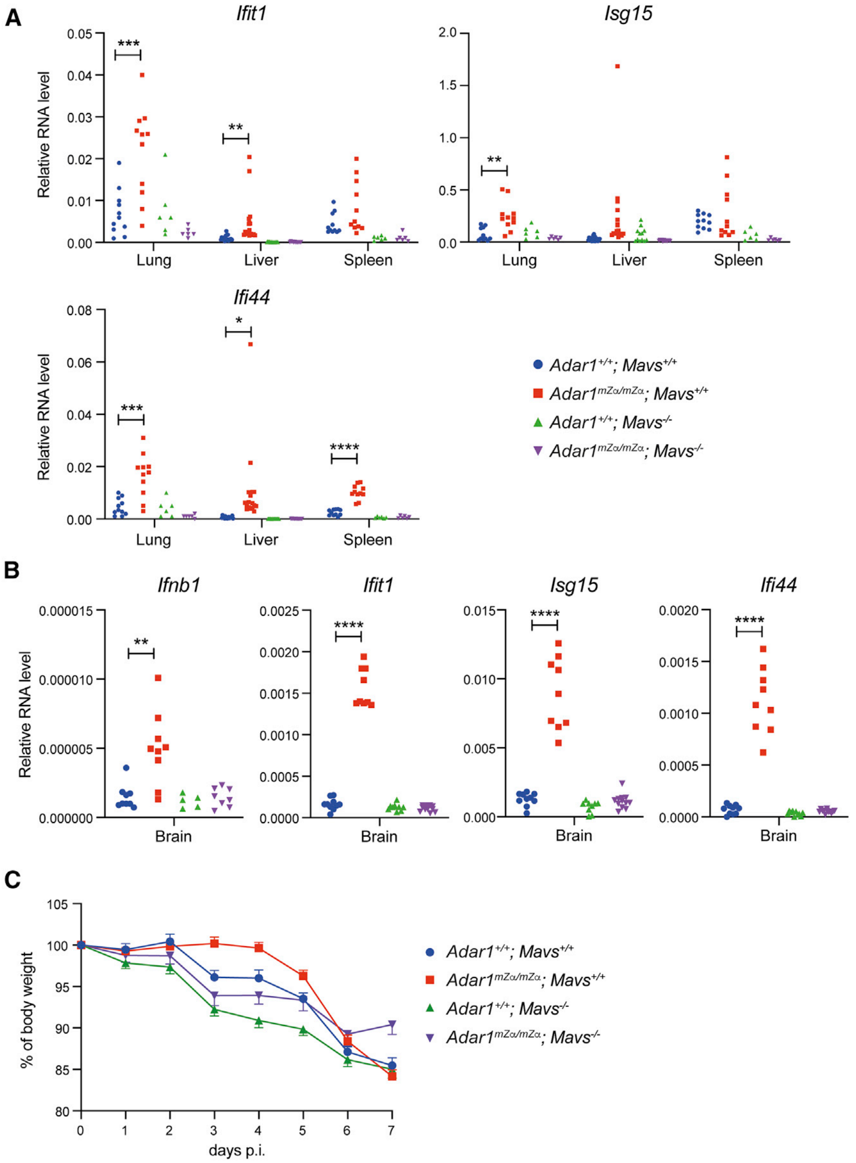

Figure 6. ISG induction in *Adar1*$^{mZ\alpha/mZ\alpha}$ mice is MAVS dependent

(A and B) Levels of the indicated mRNAs were analyzed using qRT-PCR in RNA samples extracted from tissues of WT and *Adar1*$^{mZ\alpha/mZ\alpha}$ animals that were either MAVS sufficient or deficient. Data are shown relative to *Gapdh*.

(C) Mice of the indicated genotypes were infected intranasally with 0.04 HAU of IAV strain A/X-31. Body weight was monitored daily and is shown as a percentage of starting body weight.

In (A) and (B), each dot represents an individual mouse, and pooled data from three independent experiments are shown (*p < 0.05, **p < 0.01, ***p < 0.001, and ****p < 0.0001, unpaired t test). In (C), data from two independent experiments including a total of 10–12 mice per genotype were pooled (mean with SD).

(Figure 7B). Read depth was similar between "detected" and "differential$^{WT > mZ\alpha}$" sites (Figure S7B), excluding the possibility that lack of editing in *Adar1*$^{mZ\alpha/mZ\alpha}$ samples was due to reduced sequence coverage. We then attempted to understand whether these Zα domain-dependent sites were characterized by unique properties. First, we annotated editing sites to 5′ untranslated regions (UTRs), coding sequences (CDSs), 3′ UTRs, and intronic and intergenic regions (Figure 7C). The majority of editing sites "detected" in WT samples were found in intronic regions (~83%). 3′ UTRs accounted for ~11% of edits, and intergenic regions contained ~5% of sites. Fewer than 1% of sites mapped to 5′ UTRs and CDSs. The annotation of "differential$^{WT > mZ\alpha}$" sites was similar, with a small increase in intronic sites (~86%), while 8% of "differential$^{WT > mZ\alpha}$" edits were found in 3′ UTRs.

To characterize whether the sequences surrounding Zα-dependent editing sites have a propensity to form Z-RNA, we analyzed sequences 500 nt up- and downstream of editing sites. We tested GC content, given that GC repeats have a higher tendency to adopt the Z-conformation (Davis et al., 1986). There was no enrichment of GC dinucleotides around differential$^{WT > mZ\alpha}$ edited sites compared with all detected sites (Figure S7C), and no motifs were detected in pileups of the surrounding bases. We also used the Z-hunt algorithm (Champ et al., 2004; Ho et al., 1986) to predict the likelihood of sequences around editing sites to form the Z-conformation. In parallel, we calculated the distance of editing sites to genomic regions predicted by SIBZ to form the Z-conformation (Zhabinskaya and Benham, 2011). Neither computational approach revealed differences between "detected" and "differential$^{WT > mZ\alpha}$" sites (Figures S7D and S7E). It is noteworthy that both algorithms were developed for dsDNA and may be unsuitable for predicting the Z-conformation in RNA. Future studies will be required to dissect the properties of the Zα-dependent RNA editing sites.

We also found diminished editing in WT samples at ~14% of the sites detected as edited in *Adar1*$^{mZ\alpha/mZ\alpha}$ mice (Figure 7B). These "differential$^{mZ\alpha > WT}$" sites mapped to genomic features with similar frequencies compared to "detected" and "differential$^{WT > mZ\alpha}$" sites (Figure 7C). ADAR1-p150 is encoded by an ISG and *Adar1* transcript levels increased by 1.6-fold in *Adar1*$^{mZ\alpha/mZ\alpha}$ lungs as well as in lung neutrophils and B cells (Figures S7F and S7G). Thus, it was likely that type I IFN-induced expression of ADAR1-p150 in mutant mice explained editing at these sites.

Given that many A-to-I RNA editing events occur in REs (Eisenberg and Levanon, 2018), we analyzed the enrichment of editing events in WT samples, by computing observed versus expected numbers, for each RE class (Figure 7D). This analysis showed that editing events were greatly enriched within SINEs (Figure 7D) and further showed that, with the exception of the B4 family, all SINE sub-families exhibited enrichment, albeit to

CellPress
OPEN ACCESS

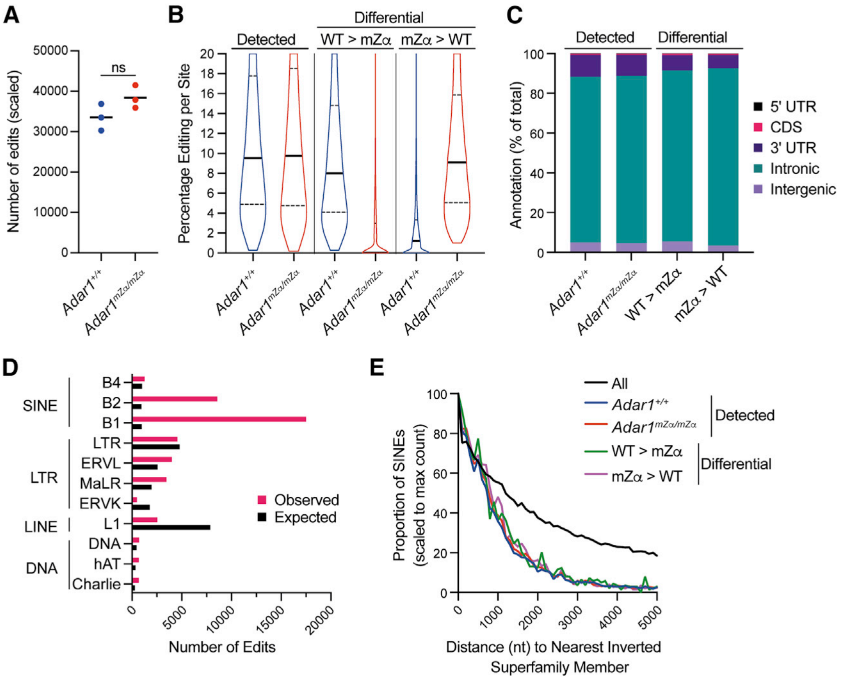

Figure 7. ADAR1-p150's Zα domain is required for editing of a subset of RNAs

(A) Editing sites were mapped in RNA sequencing reads from three WT and three *Adar1*$^{mZ\alpha/mZ\alpha}$ lung samples (Z > 2.58). The numbers of edited sites were scaled to the total number of reads per sample. Each data point corresponds to an animal, and the mean is shown (ns, not significant; unpaired t test).

(B) Editing frequencies for sites detectable in all three WT or *Adar1*$^{mZ\alpha/mZ\alpha}$ samples (left; Z > 2.58) and for differentially edited sites (right; Z > 2.58, >2-fold) are shown as violin plots. Solid horizontal lines show the median and dotted lines indicate quartiles.

(C) Editing sites detected in WT or *Adar1*$^{mZ\alpha/mZ\alpha}$ samples and differentially edited sites were matched to annotated genomic features. The percentage of sites is shown for each category.

(D) The number of expected and observed editing sites in WT samples are shown for families of REs for which either value exceeded 500. See text for details.

(E) The distances of SINEs to their nearest inverted super-family member were determined for all SINEs and for SINEs harboring an editing site detected in WT or *Adar1*$^{mZ\alpha/mZ\alpha}$ samples or containing a differentially edited site. Results are shown as proportions of SINEs with the maximum count set to 100.

See also Figure S7.

varying degrees (Figure S7H). Furthermore, there were no large-scale differences between the SINEs represented when comparing "detected" and "differential" sites (Figure S7I), suggesting that all SINEs had equal potential to contribute sites that were differentially edited between WT and *Adar1*$^{mZ\alpha/mZ\alpha}$ mice.

We next analyzed the genomic distance of edited SINEs to another SINE in inverted orientation. In human, transcripts spanning inverted repeat *Alu* elements may form duplex RNA structures recognized by ADAR1 and MDA5 (Ahmad et al., 2018; Mehdipour et al., 2020). Compared with all SINEs, we found that edited SINEs were closer to another SINE in inverted orientation (Figure 7E), although there was no difference between SINEs containing "detected" and "differential" sites. Taken together, these data demonstrated that a subset consisting of about 8% of editing sites required a functional ADAR1-p150 Zα domain for efficient A-to-I conversion.

DISCUSSION

Although Z-nucleic acids were discovered ~40 years ago, little is known to this date about their biological activities. This is due in part to their thermodynamic properties: the B- and A-conformations of dsDNA and dsRNA, respectively, are energetically favored compared with the Z-conformation, making Z-DNA and Z-RNA difficult to study (Herbert, 2019). The formation of Z-DNA may release torsional strain induced by the movement of polymerases (Wittig et al., 1992; Wölfl et al., 1995). Physiological functions of Z-RNA have remained enigmatic until recently. We and others proposed that Z-RNA is recognized by ZBP1 in settings of viral infection and autoinflammation, resulting in the induction of programmed cell death (Devos et al., 2020; Jiao et al., 2020; Maelfait et al., 2017; Sridharan et al., 2017; Wang et al., 2020; Zhang et al., 2020b).

Here, we studied ADAR1-p150, which like ZBP1 contains a Zα domain specialized in binding to Z nucleic acids. We report spontaneous induction of type I IFNs *in vivo* upon introduction of mutations into the ADAR1-p150 Zα domain that prevent binding to Z-DNA and Z-RNA. This effect was observed in multiple organs and cell types from *Adar1*$^{mZ\alpha/mZ\alpha}$ mice and required MAVS. These data showed that RIG-I-like receptors were activated by endogenously generated Z-RNAs and that editing of these Z-RNAs by ADAR1-p150 limited the response. We therefore revealed type I IFN induction as a biological function of Z-RNA.

Our computational analysis mapped ~40,000 editing sites in lung RNA samples from WT mice. These sites were enriched in SINEs but not in other classes of REs. Furthermore, we found that edited SINEs were more likely to be in proximity to another SINE in inverted orientation. These results agree with previous findings demonstrating that editing sites are enriched in non-coding sequences containing self-complementary regions predicted to form duplex RNA structures (Eisenberg and Levanon, 2018; Porath et al., 2017; Solomon et al., 2017; Tan et al., 2017).

In addition to the sites edited in WT but not mutant samples ("differential$^{WT > mZ\alpha}$"), another subset of sites was preferentially edited in *Adar1*$^{mZ\alpha/mZ\alpha}$ mice ("differential$^{mZ\alpha > WT}$"). IFN-driven increased expression of mutant ADAR1-p150 and recruitment of A-form dsRNAs via the dsRBDs may account for this observation. It is also possible that the Zα domain blocks editing at some sites. A recent study found that structural disruptions such as bulges in dsRNA substrates position ADAR1 for editing at sites 30–35 bp away from the disruption (Uzonyi et al., 2021). It will be interesting to determine in future studies whether the Zα domain plays a role in recognizing such structural disruptions.

We observed spontaneous type I IFN and ISG induction in multiple organs from *Adar1*$^{mZ\alpha/mZ\alpha}$ mice and dissected the role of different cell types in the lung. On the basis of increased

expression of ISGs such as *Ifit1* that are induced by IRF3 and IRF7, we propose that non-hematopoietic cell types as well as neutrophils initiate the IFN response in $Adar1^{mZ\alpha/mZ\alpha}$ lungs. Interestingly, a recent study showed that RNA editing increases upon intracellular acidification (Malik et al., 2021). Neutrophils produce different types of acids (Ulfig and Leichert, 2021), which may explain the induction of type I IFN responses in these cells. However, the role of neutrophils and other cell types will require validation in future studies. We were unable to reliably detect type I IFN transcripts by different qRT-PCR approaches in sorted cell samples, presumably because of their short mRNA half-life (Pasté et al., 2003; Whittemore and Maniatis, 1990). In the future, it would be interesting to develop *in situ* hybridization techniques combined with immunofluorescence to analyze IFN mRNAs in fixed or snap-frozen tissue sections with simultaneous identification of cell types.

ADAR1 mutations in human cause AGS and include the missense p.Pro193Ala mutation in the Zα domain. Interestingly, this *ADAR1* allele is common with frequencies of up to ~1/160 (www.ensembl.org) (Mannion et al., 2014). In AGS patients, homozygous p.Pro193Ala mutation is not observed; instead, this mutation occurs together with other *ADAR1* mutations (Rice et al., 2012). It is therefore likely that *ADAR1* p.Pro193Ala is hypomorphic and does not cause disease when present homozygously. Consistent with this notion, a recent study reported that $Adar1^{P195A/P195A}$ mice have no phenotype (Maurano et al., 2021). Similarly, we found that $Adar1^{mZ\alpha/mZ\alpha}$ mice did not have any gross abnormalities and were fertile. These mice nonetheless displayed type I IFN and ISG induction in multiple organs. This included the lung and bestowed protection against IAV infection at early stages. It is therefore conceivable that the *ADAR1* p.Pro193Ala variant has been maintained in humans by providing a selective advantage during viral infections due to elevated expression of antiviral factors at baseline.

It is noteworthy in this context that ADAR1 has anti- and proviral functions. ADAR1 limits or controls replication of several viruses, including measles virus, members of *Paramyxoviridae* family, IAV, HIV-1, vesicular stomatitis virus, and hepatitis delta virus (Casey, 2012; Li et al., 2010; Vogel et al., 2020; Ward et al., 2011; Weiden et al., 2014). However, for other viruses, such as Zika and Kaposi's sarcoma-associated herpesvirus, ADAR1 may facilitate replication (Zhang et al., 2020a; Zhou et al., 2019). It will be interesting for future studies to determine the role of the *ADAR1* variants unable to bind Z nucleic acids in these viral infections.

ADAR1 has recently emerged as a promising target for cancer treatment. If expressed by transformed cells *in vitro* or by tumors *in vivo*, ADAR1 protects against both cell death and anti-cancer immune responses (Gannon et al., 2018; Ishizuka et al., 2019; Liu et al., 2019). Loss of ADAR1 in cancer cells results in death or reduced growth and sensitizes to immunotherapy. Interestingly, the protective effects appear to depend on ADAR1-p150 (Gannon et al., 2018; Ishizuka et al., 2019). It is therefore possible that endogenous Z-RNAs induce anti-cancer effects upon ADAR1 loss. Future studies should test this, for example by reconstitution of ADAR1-p150 mutants unable to bind Z-RNA. Furthermore, development of inhibitors that target the Zα domain of ADAR1, the Zα-Z-RNA interaction or Z-RNA formation should be considered. Compared with deaminase inhibitors, such "Z-inhibitors" would have the advantage of specifically targeting the p150 isoform, avoiding possible detrimental consequences of targeting ADAR1-p110 (Pestal et al., 2015).

In conclusion, we discovered MAVS-dependent type I IFN induction as a biological function of Z-RNA that is curtailed by ADAR1-p150. These mechanistic insights may have implications for understanding and modulating detrimental and beneficial type I IFN responses in autoinflammation and cancer.

Limitations of study

Our goal was to address the biological function of the Zα domain and the role of Z nucleic acid recognition by ADAR1. We therefore mutated residues that are essential for Z-DNA and Z-RNA binding (Asn175 and Tyr179). In contrast, the Zα domain mutation found in some AGS patients affects Pro193 and occurs together with other mutations on the second *ADAR1* allele. This needs to be considered when extrapolating our results to human. The reader is also referred to a recent study reporting mice with a mutation at this proline (Maurano et al., 2021). Increased levels of ISG transcripts that are induced by nucleic acid sensors indicated that neutrophils initiated IFN responses in $Adar1^{mZ\alpha/mZ\alpha}$ lungs. This requires further validation, including analysis of neutrophils in $Adar1^{mZ\alpha/mZ\alpha}$; $Ifnar^{-/-}$ and *Ly6G-Cre*; $Adar1^{fl\text{-}mZ\alpha/fl\text{-}mZ\alpha}$ mice and RNA-seq of purified neutrophils. Finally, we did not delineate specific properties of Zα-dependent editing sites. Analysis of editing in cell types and future computational study, for example of local RNA folding, may provide further insight.

STAR★METHODS

Detailed methods are provided in the online version of this paper and include the following:

- KEY RESOURCES TABLE
- RESOURCE AVAILABILITY
  - Lead contact
  - Materials availability
  - Data and code availability
- EXPERIMENTAL MODEL AND SUBJECT DETAILS
  - Mice
  - Cells
- METHOD DETAILS
  - RNA extraction and RT-qPCR
  - *In vivo* infection
  - Median tissue culture infective dose ($TCID_{50}$) assay
  - Western blot
  - Flow cytometry
  - Magnetic cell fraction
  - Generation of bone marrow chimeric animals
  - ELISA
  - RNA-seq and data processing
  - Differential expression analysis
  - Detection of A-to-I editing
  - Analysis of A-to-I editing sites
- QUANTIFICATION AND STATISTICAL ANALYSIS

SUPPLEMENTAL INFORMATION

Supplemental information can be found online at https://doi.org/10.1016/j.immuni.2021.08.011.

CellPress
OPEN ACCESS

ACKNOWLEDGMENTS

We thank P. Shing Ho and Craig Benham for providing access to the Z-hunt and SIBZ codes, respectively, and for their helpful correspondence. We further thank Daniel Stetson, Andrew Oberst, Jonathan Maelfait, Caetano Reis e Sousa, David Ron, Annemarthe van Der Veen, Irina Udalova, Erinke van Grinsven, and members of the Rehwinkel lab for discussion. We thank Ziqi Long and Oliver Bannard for their help with generating BM chimeras and Hal Drakesmith for support of the IAV work. This work was funded by the U.K. Medical Research Council (MRC) (MRC core funding of the MRC Human Immunology Unit to J.R.), the Wellcome Trust (grant 100954 to J.R.), the Lister Institute (to J.R.), and the Francis Crick Institute (to G.K.), which receives its core funding from Cancer Research UK, the MRC, and the Wellcome Trust. T.K.T. was funded by the Townsend-Jeantet Charitable Trust (charity number 1011770) and the EPA Cephalosporin Early Career Researcher Fund. The funders had no role in study design, data collection and analysis, decision to publish, or preparation of the manuscript.

AUTHOR CONTRIBUTIONS

Conceptualization, Q.T. and J.R.; Methodology, Q.T., R.E.R., and G.Y.; Software, R.E.R. and G.Y.; Validation, Q.T. and J.R.; Formal Analysis, Q.T., R.E.R., G.Y., and J.R.; Investigation, Q.T., R.E.R., G.Y., A.K.H., T.D., T.K.T., and A.B.; Resources, A.T. and G.K.; Data Curation, Q.T., R.E.R., and G.Y.; Writing – Original Draft, Q.T., G.Y., and J.R.; Writing – Review & Editing, all authors; Visualization, Q.T., R.E.R., G.Y., and J.R.; Supervision, J.R., A.T., and G.K.; Project Administration, Q.T.; Funding Acquisition, J.R.

DECLARATION OF INTERESTS

The authors declare no competing interests.

Received: December 4, 2020
Revised: May 13, 2021
Accepted: August 11, 2021
Published: September 14, 2021

REFERENCES

Ahmad, S., Mu, X., Yang, F., Greenwald, E., Park, J.W., Jacob, E., Zhang, C.Z., and Hur, S. (2018). Breaching self-tolerance to Alu duplex RNA underlies MDA5-mediated inflammation. Cell *172*, 797–810.e13.

Amemiya, H.M., Kundaje, A., and Boyle, A.P. (2019). The ENCODE Blacklist: identification of problematic regions of the genome. Sci. Rep. *9*, 9354.

Ashley, C.L., Abendroth, A., McSharry, B.P., and Slobedman, B. (2019). Interferon-independent upregulation of interferon-stimulated genes during human cytomegalovirus infection is dependent on IRF3 expression. Viruses *11*, E246.

Athanasiadis, A. (2012). Zalpha-domains: at the intersection between RNA editing and innate immunity. Semin. Cell Dev. Biol. *23*, 275–280.

Athanasiadis, A., Placido, D., Maas, S., Brown, B.A., 2nd, Lowenhaupt, K., and Rich, A. (2005). The crystal structure of the Zbeta domain of the RNA-editing enzyme ADAR1 reveals distinct conserved surfaces among Z-domains. J. Mol. Biol. *351*, 496–507.

Attig, J., Young, G.R., Stoye, J.P., and Kassiotis, G. (2017). Physiological and pathological transcriptional activation of endogenous retroelements assessed by RNA-sequencing of B lymphocytes. Front. Microbiol. *8*, 2489.

Bajad, P., Ebner, F., Amman, F., Szabó, B., Kapoor, U., Manjali, G., Hildebrandt, A., Janisiw, M.P., and Jantsch, M.F. (2020). An internal deletion of ADAR rescued by MAVS deficiency leads to a minute phenotype. Nucleic Acids Res. *48*, 3286–3303.

Bartok, E., and Hartmann, G. (2020). Immune sensing mechanisms that discriminate self from altered self and foreign nucleic acids. Immunity *53*, 54–77.

Brown, B.A., 2nd, Lowenhaupt, K., Wilbert, C.M., Hanlon, E.B., and Rich, A. (2000). The zalpha domain of the editing enzyme dsRNA adenosine deaminase binds left-handed Z-RNA as well as Z-DNA. Proc. Natl. Acad. Sci. U S A *97*, 13532–13536.

Casey, J.L. (2012). Control of ADAR1 editing of hepatitis delta virus RNAs. Curr. Top. Microbiol. Immunol. *353*, 123–143.

Cavalcante, R.G., and Sartor, M.A. (2017). annotatr: genomic regions in context. Bioinformatics *33*, 2381–2383.

Champ, P.C., Maurice, S., Vargason, J.M., Camp, T., and Ho, P.S. (2004). Distributions of Z-DNA and nuclear factor I in human chromosome 22: a model for coupled transcriptional regulation. Nucleic Acids Res. *32*, 6501–6510.

Chung, H., Calis, J.J.A., Wu, X., Sun, T., Yu, Y., Sarbanes, S.L., Dao Thi, V.L., Shilvock, A.R., Hoffmann, H.H., Rosenberg, B.R., and Rice, C.M. (2018). Human ADAR1 prevents endogenous RNA from triggering translational shutdown. Cell *172*, 811–824.e14.

Chuong, E.B., Elde, N.C., and Feschotte, C. (2016). Regulatory evolution of innate immunity through co-option of endogenous retroviruses. Science *351*, 1083–1087.

Davis, P.W., Hall, K., Cruz, P., Tinoco, I., Jr., and Neilson, T. (1986). The tetraribonucleotide rCpGpCpG forms a left-handed Z-RNA double-helix. Nucleic Acids Res. *14*, 1279–1291.

DeFilippis, V.R., Robinson, B., Keck, T.M., Hansen, S.G., Nelson, J.A., and Früh, K.J. (2006). Interferon regulatory factor 3 is necessary for induction of antiviral genes during human cytomegalovirus infection. J. Virol. *80*, 1032–1037.

Devos, M., Tanghe, G., Gilbert, B., Dierick, E., Verheirstraeten, M., Nemegeer, J., de Reuver, R., Lefebvre, S., De Munck, J., Rehwinkel, J., et al. (2020). Sensing of endogenous nucleic acids by ZBP1 induces keratinocyte necroptosis and skin inflammation. J. Exp. Med. *217*, e20191913.

Di Tommaso, P., Chatzou, M., Floden, E.W., Barja, P.P., Palumbo, E., and Notredame, C. (2017). Nextflow enables reproducible computational workflows. Nat. Biotechnol. *35*, 316–319.

Dias, A.G., Jr., Sampaio, N.G., and Rehwinkel, J. (2019). A balancing act: MDA5 in antiviral immunity and autoinflammation. Trends Microbiol. *27*, 75–85.

Dobin, A., Davis, C.A., Schlesinger, F., Drenkow, J., Zaleski, C., Jha, S., Batut, P., Chaisson, M., and Gingeras, T.R. (2013). STAR: ultrafast universal RNA-seq aligner. Bioinformatics *29*, 15–21.

Eisenberg, E., and Levanon, E.Y. (2018). A-to-I RNA editing—immune protector and transcriptome diversifier. Nat. Rev. Genet. *19*, 473–490.

Feng, S., Li, H., Zhao, J., Pervushin, K., Lowenhaupt, K., Schwartz, T.U., and Dröge, P. (2011). Alternate rRNA secondary structures as regulators of translation. Nat. Struct. Mol. Biol. *18*, 169–176.

Gannon, H.S., Zou, T., Kiessling, M.K., Gao, G.F., Cai, D., Choi, P.S., Ivan, A.P., Buchumenski, I., Berger, A.C., Goldstein, J.T., et al. (2018). Identification of ADAR1 adenosine deaminase dependency in a subset of cancer cells. Nat. Commun. *9*, 5450.

Gel, B., Díez-Villanueva, A., Serra, E., Buschbeck, M., Peinado, M.A., and Malinverni, R. (2016). regioneR: an R/Bioconductor package for the association analysis of genomic regions based on permutation tests. Bioinformatics *32*, 289–291.

Goubau, D., Romieu-Mourez, R., Solis, M., Hernandez, E., Mesplède, T., Lin, R., Leaman, D., and Hiscott, J. (2009). Transcriptional re-programming of primary macrophages reveals distinct apoptotic and anti-tumoral functions of IRF-3 and IRF-7. Eur. J. Immunol. *39*, 527–540.

Grandvaux, N., Servant, M.J., tenOever, B., Sen, G.C., Balachandran, S., Barber, G.N., Lin, R., and Hiscott, J. (2002). Transcriptional profiling of interferon regulatory factor 3 target genes: direct involvement in the regulation of interferon-stimulated genes. J. Virol. *76*, 5532–5539.

Guallar, D., Fuentes-Iglesias, A., Souto, Y., Ameneiro, C., Freire-Agulleiro, O., Pardavila, J.A., Escudero, A., Garcia-Outeiral, V., Moreira, T., Saenz, C., et al. (2020). ADAR1-dependent RNA editing promotes MET and iPSC reprogramming by alleviating ER stress. Cell Stem Cell *27*, 300–314.e11.

Hall, K., Cruz, P., Tinoco, I., Jr., Jovin, T.M., and van de Sande, J.H. (1984). 'Z-RNA'—a left-handed RNA double helix. Nature *311*, 584–586.

Harding, H.P., Zhang, Y., Zeng, H., Novoa, I., Lu, P.D., Calfon, M., Sadri, N., Yun, C., Popko, B., Paules, R., et al. (2003). An integrated stress response regulates amino acid metabolism and resistance to oxidative stress. Mol. Cell *11*, 619–633.

Hartner, J.C., Schmittwolf, C., Kispert, A., Müller, A.M., Higuchi, M., and Seeburg, P.H. (2004). Liver disintegration in the mouse embryo caused by deficiency in the RNA-editing enzyme ADAR1. J. Biol. Chem. *279*, 4894–4902.

Hartner, J.C., Walkley, C.R., Lu, J., and Orkin, S.H. (2009). ADAR1 is essential for the maintenance of hematopoiesis and suppression of interferon signaling. Nat. Immunol. *10*, 109–115.

Heraud-Farlow, J.E., and Walkley, C.R. (2020). What do editors do? Understanding the physiological functions of A-to-I RNA editing by adenosine deaminase acting on RNAs. Open Biol. *10*, 200085.

Herbert, A. (2019). Z-DNA and Z-RNA in human disease. Commun. Biol. *2*, 7.

Ho, P.S., Ellison, M.J., Quigley, G.J., and Rich, A. (1986). A computer aided thermodynamic approach for predicting the formation of Z-DNA in naturally occurring sequences. EMBO J. *5*, 2737–2744.

Ishizuka, J.J., Manguso, R.T., Cheruiyot, C.K., Bi, K., Panda, A., Iracheta-Vellve, A., Miller, B.C., Du, P.P., Yates, K.B., Dubrot, J., et al. (2019). Loss of ADAR1 in tumours overcomes resistance to immune checkpoint blockade. Nature *565*, 43–48.

Jiao, H., Wachsmuth, L., Kumari, S., Schwarzer, R., Lin, J., Eren, R.O., Fisher, A., Lane, R., Young, G.R., Kassiotis, G., et al. (2020). Z-nucleic-acid sensing triggers ZBP1-dependent necroptosis and inflammation. Nature *580*, 391–395.

Kesavardhana, S., and Kanneganti, T.D. (2020). ZBP1: A STARGÅTE to decode the biology of Z-nucleic acids in disease. J. Exp. Med. *217*, e20200885.

Kim, D., Paggi, J.M., Park, C., Bennett, C., and Salzberg, S.L. (2019). Graph-based genome alignment and genotyping with HISAT2 and HISAT-genotype. Nat. Biotechnol. *37*, 907–915.

Kim, S.H., Lim, S.H., Lee, A.R., Kwon, D.H., Song, H.K., Lee, J.H., Cho, M., Johner, A., Lee, N.K., and Hong, S.C. (2018). Unveiling the pathway to Z-DNA in the protein-induced B-Z transition. Nucleic Acids Res. *46*, 4129–4137.

Kim, Y.G., Muralinath, M., Brandt, T., Pearcy, M., Hauns, K., Lowenhaupt, K., Jacobs, B.L., and Rich, A. (2003). A role for Z-DNA binding in vaccinia virus pathogenesis. Proc. Natl. Acad. Sci. U S A *100*, 6974–6979.

Lazear, H.M., Lancaster, A., Wilkins, C., Suthar, M.S., Huang, A., Vick, S.C., Clepper, L., Thackray, L., Brassil, M.M., Virgin, H.W., et al. (2013). IRF-3, IRF-5, and IRF-7 coordinately regulate the type I IFN response in myeloid dendritic cells downstream of MAVS signaling. PLoS Pathog. *9*, e1003118.

Li, H. (2011). A statistical framework for SNP calling, mutation discovery, association mapping and population genetical parameter estimation from sequencing data. Bioinformatics *27*, 2987–2993.

Li, H., Xiao, J., Li, J., Lu, L., Feng, S., and Dröge, P. (2009). Human genomic Z-DNA segments probed by the Z alpha domain of ADAR1. Nucleic Acids Res. *37*, 2737–2746.

Li, X.D., Wu, J., Gao, D., Wang, H., Sun, L., and Chen, Z.J. (2013). Pivotal roles of cGAS-cGAMP signaling in antiviral defense and immune adjuvant effects. Science *341*, 1390–1394.

Li, Y., Banerjee, S., Goldstein, S.A., Dong, B., Gaughan, C., Rath, S., Donovan, J., Korennykh, A., Silverman, R.H., and Weiss, S.R. (2017). Ribonuclease L mediates the cell-lethal phenotype of double-stranded RNA editing enzyme ADAR1 deficiency in a human cell line. eLife *6*, e25687.

Li, Z., Wolff, K.C., and Samuel, C.E. (2010). RNA adenosine deaminase ADAR1 deficiency leads to increased activation of protein kinase PKR and reduced vesicular stomatitis virus growth following interferon treatment. Virology *396*, 316–322.

Liao, Y., Smyth, G.K., and Shi, W. (2014). featureCounts: an efficient general purpose program for assigning sequence reads to genomic features. Bioinformatics *30*, 923–930.

Liddicoat, B.J., Piskol, R., Chalk, A.M., Ramaswami, G., Higuchi, M., Hartner, J.C., Li, J.B., Seeburg, P.H., and Walkley, C.R. (2015). RNA editing by ADAR1 prevents MDA5 sensing of endogenous dsRNA as nonself. Science *349*, 1115–1120.

Liu, H., Golji, J., Brodeur, L.K., Chung, F.S., Chen, J.T., deBeaumont, R.S., Bullock, C.P., Jones, M.D., Kerr, G., Li, L., et al. (2019). Tumor-derived IFN triggers chronic pathway agonism and sensitivity to ADAR loss. Nat. Med. *25*, 95–102.

Love, M.I., Huber, W., and Anders, S. (2014). Moderated estimation of fold change and dispersion for RNA-seq data with DESeq2. Genome Biol. *15*, 550.

Luo, H., Wang, D., Che, H.L., Zhao, Y., and Jin, H. (2012). Pathological observations of lung inflammation after administration of IP-10 in influenza virus- and respiratory syncytial virus-infected mice. Exp. Ther. Med. *3*, 76–79.

Maelfait, J., Liverpool, L., Bridgeman, A., Ragan, K.B., Upton, J.W., and Rehwinkel, J. (2017). Sensing of viral and endogenous RNA by ZBP1/DAI induces necroptosis. EMBO J. *36*, 2529–2543.

Maelfait, J., Liverpool, L., and Rehwinkel, J. (2020). Nucleic acid sensors and programmed cell death. J. Mol. Biol. *432*, 552–568.

Malik, T.N., Doherty, E.E., Gaded, V.M., Hill, T.M., Beal, P.A., and Emeson, R.B. (2021). Regulation of RNA editing by intracellular acidification. Nucleic Acids Res. *49*, 4020–4036.

Mannion, N.M., Greenwood, S.M., Young, R., Cox, S., Brindle, J., Read, D., Nellåker, C., Vesely, C., Ponting, C.P., McLaughlin, P.J., et al. (2014). The RNA-editing enzyme ADAR1 controls innate immune responses to RNA. Cell Rep. *9*, 1482–1494.

Martin, M. (2011). Cutadapt removes adapter sequences from high-throughput sequencing reads. EMBnet.journal *17*, 3.

Maurano, M., Snyder, J.M., Connelly, C., Henao-Mejia, J., Sidrauski, C., and Stetson, D.B. (2021). Protein kinase R and the integrated stress response drive immunopathology caused by mutations in the RNA deaminase ADAR1. Immunity. In this issue. https://doi.org/10.1016/j.immuni.2021.07.001.

Mehdipour, P., Marhon, S.A., Ettayebi, I., Chakravarthy, A., Hosseini, A., Wang, Y., de Castro, F.A., Loo Yau, H., Ishak, C., Abelson, S., et al. (2020). Epigenetic therapy induces transcription of inverted SINEs and ADAR1 dependency. Nature *588*, 169–173.

Michallet, M.C., Meylan, E., Ermolaeva, M.A., Vazquez, J., Rebsamen, M., Curran, J., Poeck, H., Bscheider, M., Hartmann, G., König, M., et al. (2008). TRADD protein is an essential component of the RIG-like helicase antiviral pathway. Immunity *28*, 651–661.

Pakos-Zebrucka, K., Koryga, I., Mnich, K., Ljujic, M., Samali, A., and Gorman, A.M. (2016). The integrated stress response. EMBO Rep. *17*, 1374–1395.

Pasté, M., Huez, G., and Kruys, V. (2003). Deadenylation of interferon-beta mRNA is mediated by both the AU-rich element in the 3′-untranslated region and an instability sequence in the coding region. Eur. J. Biochem. *270*, 1590–1597.

Perng, Y.C., and Lenschow, D.J. (2018). ISG15 in antiviral immunity and beyond. Nat. Rev. Microbiol. *16*, 423–439.

Pestal, K., Funk, C.C., Snyder, J.M., Price, N.D., Treuting, P.M., and Stetson, D.B. (2015). Isoforms of RNA-editing enzyme ADAR1 independently control nucleic acid sensor MDA5-driven autoimmunity and multi-organ development. Immunity *43*, 933–944.

Pfaller, C.K., Donohue, R.C., Nersisyan, S., Brodsky, L., and Cattaneo, R. (2018). Extensive editing of cellular and viral double-stranded RNA structures accounts for innate immunity suppression and the proviral activity of ADAR1p150. PLoS Biol. *16*, e2006577.

Pichlmair, A., Lassnig, C., Eberle, C.A., Górna, M.W., Baumann, C.L., Burkard, T.R., Bürckstümmer, T., Stefanovic, A., Krieger, S., Bennett, K.L., et al. (2011). IFIT1 is an antiviral protein that recognizes 5′-triphosphate RNA. Nat. Immunol. *12*, 624–630.

Piechotta, M., and Dieterich, C. (2013). ACCUSA2: multi-purpose SNV calling enhanced by probabilistic integration of quality scores. Bioinformatics *29*, 1809–1810.

Piechotta, M., Wyler, E., Ohler, U., Landthaler, M., and Dieterich, C. (2017). JACUSA: site-specific identification of RNA editing events from replicate sequencing data. BMC Bioinformatics *18*, 7.

CellPress
OPEN ACCESS

Porath, H.T., Knisbacher, B.A., Eisenberg, E., and Levanon, E.Y. (2017). Massive A-to-I RNA editing is common across the Metazoa and correlates with dsRNA abundance. Genome Biol. *18*, 185.

Powell, T.J., Rijal, P., McEwen-Smith, R.M., Byun, H., Hardwick, M., Schimanski, L.M., Huang, K.A., Daniels, R.S., and Townsend, A.R.M. (2019). A single cycle influenza virus coated in H7 haemagglutinin generates neutralizing antibody responses to haemagglutinin and neuraminidase glycoproteins and protection from heterotypic challenge. J. Gen. Virol. *100*, 431–445.

Powell, T.J., Silk, J.D., Sharps, J., Fodor, E., and Townsend, A.R. (2012). Pseudotyped influenza A virus as a vaccine for the induction of heterotypic immunity. J. Virol. *86*, 13397–13406.

Quinlan, A.R., and Hall, I.M. (2010). BEDTools: a flexible suite of utilities for comparing genomic features. Bioinformatics *26*, 841–842.

Reed, L.J., and Muench, H. (1938). A simple method of estimating fifty per cent endpoints12. Am. J. Epidemiol. *27*, 493–497.

Reich, D.P., and Bass, B.L. (2019). Mapping the dsRNA World. Cold Spring Harb. Perspect. Biol. *11*, a035352.

Rice, G.I., Kasher, P.R., Forte, G.M., Mannion, N.M., Greenwood, S.M., Szynkiewicz, M., Dickerson, J.E., Bhaskar, S.S., Zampini, M., Briggs, T.A., et al. (2012). Mutations in ADAR1 cause Aicardi-Goutières syndrome associated with a type I interferon signature. Nat. Genet. *44*, 1243–1248.

Rigby, R.E., Wise, H.M., Smith, N., Digard, P., and Rehwinkel, J. (2019). PA-X antagonises MAVS-dependent accumulation of early type I interferon messenger RNAs during influenza A virus infection. Sci. Rep. *9*, 7216.

Schade, M., Turner, C.J., Lowenhaupt, K., Rich, A., and Herbert, A. (1999). Structure-function analysis of the Z-DNA-binding domain Zalpha of dsRNA adenosine deaminase type I reveals similarity to the (alpha + beta) family of helix-turn-helix proteins. EMBO J. *18*, 470–479.

Schoggins, J.W. (2019). Interferon-stimulated genes: what do they all do? Annu. Rev. Virol. *6*, 567–584.

Schwartz, T., Rould, M.A., Lowenhaupt, K., Herbert, A., and Rich, A. (1999). Crystal structure of the Zalpha domain of the human editing enzyme ADAR1 bound to left-handed Z-DNA. Science *284*, 1841–1845.

Solomon, O., Di Segni, A., Cesarkas, K., Porath, H.T., Marcu-Malina, V., Mizrahi, O., Stern-Ginossar, N., Kol, N., Farage-Barhom, S., Glick-Saar, E., et al. (2017). RNA editing by ADAR1 leads to context-dependent transcriptome-wide changes in RNA secondary structure. Nat. Commun. *8*, 1440.

Song, Y., Yang, W., Fu, Q., Wu, L., Zhao, X., Zhang, Y., and Zhang, R. (2020). irCLASH reveals RNA substrates recognized by human ADARs. Nat. Struct. Mol. Biol. *27*, 351–362.

Sridharan, H., Ragan, K.B., Guo, H., Gilley, R.P., Landsteiner, V.J., Kaiser, W.J., and Upton, J.W. (2017). Murine cytomegalovirus IE3-dependent transcription is required for DAI/ZBP1-mediated necroptosis. EMBO Rep. *18*, 1429–1441.

Tan, M.H., Li, Q., Shanmugam, R., Piskol, R., Kohler, J., Young, A.N., Liu, K.I., Zhang, R., Ramaswami, G., Ariyoshi, K., et al.; GTEx Consortium; Laboratory, Data Analysis &Coordinating Center (LDACC)—Analysis Working Group; Statistical Methods groups—Analysis Working Group; Enhancing GTEx (eGTEx) groups; NIH Common Fund; NIH/NCI; NIH/NHGRI; NIH/NIMH; NIH/NIDA; Biospecimen Collection Source Site—NDRI; Biospecimen Collection Source Site—RPCI; Biospecimen Core Resource—VARI; Brain Bank Repository—University of Miami Brain Endowment Bank; Leidos Biomedical—Project Management; ELSI Study; Genome Browser Data Integration &Visualization—EBI; Genome Browser Data Integration &Visualization—UCSC Genomics Institute, University of California Santa Cruz (2017). Dynamic landscape and regulation of RNA editing in mammals. Nature 550, 249–254.

Uggenti, C., Lepelley, A., and Crow, Y.J. (2019). Self-awareness: nucleic acid-driven inflammation and the type I interferonopathies. Annu. Rev. Immunol. *37*, 247–267.

Ulfig, A., and Leichert, L.I. (2021). The effects of neutrophil-generated hypochlorous acid and other hypohalous acids on host and pathogens. Cell. Mol. Life Sci. *78*, 385–414.

Uzonyi, A., Nir, R., Shliefer, O., Stern-Ginossar, N., Antebi, Y., Stelzer, Y., Levanon, E.Y., and Schwartz, S. (2021). Deciphering the principles of the RNA editing code via large-scale systematic probing. Mol. Cell *81*, 2374–2387.e3.

Vogel, O.A., Han, J., Liang, C.Y., Manicassamy, S., Perez, J.T., and Manicassamy, B. (2020). The p150 isoform of ADAR1 blocks sustained RLR signaling and apoptosis during influenza virus infection. PLoS Pathog. *16*, e1008842.

Wang, A.H., Quigley, G.J., Kolpak, F.J., Crawford, J.L., van Boom, J.H., van der Marel, G., and Rich, A. (1979). Molecular structure of a left-handed double helical DNA fragment at atomic resolution. Nature *282*, 680–686.

Wang, L., Wang, S., and Li, W. (2012). RSeQC: quality control of RNA-seq experiments. Bioinformatics *28*, 2184–2185.

Wang, Q., Miyakoda, M., Yang, W., Khillan, J., Stachura, D.L., Weiss, M.J., and Nishikura, K. (2004). Stress-induced apoptosis associated with null mutation of ADAR1 RNA editing deaminase gene. J. Biol. Chem. *279*, 4952–4961.

Wang, R., Li, H., Wu, J., Cai, Z.Y., Li, B., Ni, H., Qiu, X., Chen, H., Liu, W., Yang, Z.H., et al. (2020). Gut stem cell necroptosis by genome instability triggers bowel inflammation. Nature *580*, 386–390.

Ward, S.V., George, C.X., Welch, M.J., Liou, L.Y., Hahm, B., Lewicki, H., de la Torre, J.C., Samuel, C.E., and Oldstone, M.B. (2011). RNA editing enzyme adenosine deaminase is a restriction factor for controlling measles virus replication that also is required for embryogenesis. Proc. Natl. Acad. Sci. U S A *108*, 331–336.

Weiden, M.D., Hoshino, S., Levy, D.N., Li, Y., Kumar, R., Burke, S.A., Dawson, R., Hioe, C.E., Borkowsky, W., Rom, W.N., and Hoshino, Y. (2014). Adenosine deaminase acting on RNA-1 (ADAR1) inhibits HIV-1 replication in human alveolar macrophages. PLoS ONE *9*, e108476.

Whittemore, L.A., and Maniatis, T. (1990). Postinduction turnoff of beta-interferon gene expression. Mol. Cell. Biol. *10*, 1329–1337.

Wittig, B., Wölfl, S., Dorbic, T., Vahrson, W., and Rich, A. (1992). Transcription of human c-myc in permeabilized nuclei is associated with formation of Z-DNA in three discrete regions of the gene. EMBO J. *11*, 4653–4663.

Wölfl, S., Wittig, B., and Rich, A. (1995). Identification of transcriptionally induced Z-DNA segments in the human c-myc gene. Biochim. Biophys. Acta *1264*, 294–302.

Young, M.D., Wakefield, M.J., Smyth, G.K., and Oshlack, A. (2010). Gene Ontology analysis for RNA-seq: accounting for selection bias. Genome Biol. *11*, R14.

Zhabinskaya, D., and Benham, C.J. (2011). Theoretical analysis of the stress induced B-Z transition in superhelical DNA. PLoS Comput. Biol. *7*, e1001051.

Zhang, H., Ni, G., and Damania, B. (2020a). ADAR1 facilitates KSHV lytic reactivation by modulating the RLR-dependent signaling pathway. Cell Rep. *31*, 107564.

Zhang, T., Yin, C., Boyd, D.F., Quarato, G., Ingram, J.P., Shubina, M., Ragan, K.B., Ishizuka, T., Crawford, J.C., Tummers, B., et al. (2020b). Influenza virus Z-RNAs induce ZBP1-mediated necroptosis. Cell *180*, 1115–1129.e13.

Zhou, S., Yang, C., Zhao, F., Huang, Y., Lin, Y., Huang, C., Ma, X., Du, J., Wang, Y., Long, G., et al. (2019). Double-stranded RNA deaminase ADAR1 promotes the Zika virus replication by inhibiting the activation of protein kinase PKR. J. Biol. Chem. *294*, 18168–18180.

STAR★METHODS

KEY RESOURCES TABLE

REAGENT or RESOURCE	SOURCE	IDENTIFIER
**Antibodies**		
Anti-ADAR1	Santa Cruz	Cat# sc-73408; RRID: AB_2222767
Anti-CD45.1-AF488 (clone A20)	Biolegend	Cat# 110718; RRID: AB_492862
Anti-CD45.2-AF488 (clone 104)	Biolegend	Cat# 109815; RRID: AB_492869
Anti-CD45.2-PE (clone 104)	Biolegend	Cat# 109808; RRID: AB_313445
Anti-CD11c-APC (clone N418)	eBioscience	Cat# 17-0114-81; RRID: AB_469345
Anti-CD11b-BV785 (clone M1/70)	Biolegend	Cat# 101243; RRID: AB_2561373
Anti-MHCII-AF700 (Clone M5/114.15.2)	eBioscience	Cat# 56-5321-80; RRID: AB_494010
Anti-CD24-BV605 (Clone M1/69)	Biolegend	Cat# 101827; RRID: AB_2563464
Anti-CD64-PE (Clone X54-5/7.1)	Biolegend	Cat# 139304; RRID: AB_10612740
Anti-MHCII-e780 (Clone M5/114.15.2)	eBioscience	Cat# 47-5321-82; RRID: AB_1548783
Anti-CD31-PEcy7 (Clone 390)	eBioscience	Cat# 25-0311-81; RRID: AB_2734962
Anti-CD326-APC (Clone G8.8)	eBioscience	Cat# 17-5791-80; RRID: AB_2734965
Anti-ly6G-AF700 (Clone 1A8)	Biolegend	Cat# 127622; RRID: AB_10643269
Anti-siglecF-BV421 (Clone E50-2440)	BD	Cat# 565934; RRID: AB_2739398
**Bacterial and virus strains**		
Influenza A virus (A/X-31; A/HongKong/1/1968)	Laboratory of Alain R. Townsend	(Powell et al., 2019)
**Chemicals, peptides, and recombinant proteins**		
Collagenase, type II	Worthington Biochemical	Cat# LS004204
Dnase I	Sigma Aldrich	Cat# D4263
LIVE/DEAD Fixable Aqua Dead Cell Stain Kit	Invitrogen	Cat# L34966
Recombinant mouse GM-CSF	Peprotech	Cat# 315-03
**Critical commercial assays**		
RNeasy Plus Kit	QIAGEN	Cat# 74134
Mouse IL-6 Uncoated ELISA Kit	Life technology	Cat# 88-7064-88
Taqman Gene expression master mix	Life technology	Cat# 4304437
**Deposited data**		
RNaseq analysis of WT and *Adar1*$^{mZ\alpha/mZ\alpha}$ mice	ENA	PRJEB45231
**Experimental models: Cell lines**		
MDCK-SIAT cells for Influenza virus propagation	ECACC	Cat# 05071502
**Experimental models: Organisms/strains**		
C57BL/6 mice	Envigo and University of Oxford Biomedical Services	N/A
Adar1$^{mZ\alpha/mZ\alpha}$ mice	This paper	N/A
B6SJLCD45.1 mice	University of Oxford Biomedical Services	N/A
Mavs$^{-/-}$ mice	Laboratory of J. Tschopp	Michallet et al., 2008
**Oligonucleotides**		
Genotyping primer F 5′-TGACGAGA GACTTGTTTTCCTAGCATG-3′	Sigma	N/A

(Continued on next page)

CellPress
OPEN ACCESS

Continued

REAGENT or RESOURCE	SOURCE	IDENTIFIER
Genotyping primer R1 5′-TGCCTCAATGA GACCTCCAACTTAACTC-3′	Sigma	N/A
Genotyping prime R2^{WT} 5′-CAG GGAGTACAAAATACGATT-3′	Sigma	N/A
Genotyping prime R2^{MUT} 5′-CAG GGAGGCCAAAATACGAGC-3′	Sigma	N/A
Taqman probes and qPCR primers, see Table S2	This paper	N/A
Software and algorithms		
GraphPad Prism v8	GraphPad Software	http://www.graphpad.com

RESOURCE AVAILABILITY

Lead contact

Further information and requests for resources and reagents should be directed to and will be fulfilled by the lead contact, Jan Rehwinkel (jan.rehwinkel@imm.ox.ac.uk).

Materials availability

All unique reagents generated in this study are available from the Lead Contact with a completed Materials Transfer Agreement.

Data and code availability

RNA-seq data have been deposited at ENA and are publicly available as of the date of publication. The accession number is listed in the key resources table. All original code has been deposited at GitHub [github.com/A-N-Other/pedestal, commit 2c55bf3] and is publicly available as of the date of publication. Any additional information required to reanalyse the data reported in this paper is available from the lead contact upon request.

EXPERIMENTAL MODEL AND SUBJECT DETAILS

Mice

Mice were housed and bred under standard conditions at the University of Oxford Biomedical Services Animal Facilities. All mice were on the C57BL/6 background and were 8-10 week old; male and female animals were used. This work was performed in the animal facilities at the University of Oxford, in accordance with the UK Animal (Scientific Procedures) Act 1986 and institutional guidelines for animal care. This work was approved by project licenses granted by the UK Home Office (PPL numbers PC041D0AB, PBA43A2E4 and P79A4C5BA) and was also approved by the Institutional Animal Ethics Committee Review Board at the University of Oxford.

Adar1$^{+/fl\text{-}mZ\alpha}$ mice were generated by Cyagen. In brief, genomic fragments containing homology arms were amplified from a BAC and were sequentially assembled into a targeting vector together with recombination sites and selection markers as shown in Figure S1A. Successful assembly of the targeting vector was verified by restriction digest and sequencing. The linearized vector was subsequently delivered to ES cells (C57BL/6) via electroporation, followed by drug selection, PCR screening and sequencing. After confirming correctly targeted ES clones via Southern blotting, we selected clones for blastocyst microinjection, followed by chimera production. Founders were confirmed as germline-transmitted via crossbreeding with WT animals. The Neo cassette was flanked by Rox sites and contained a Dre recombinase controlled by a promoter active in the germline, resulting in deletion of the Neo cassette in F1 animals (Figure S1A). These *Adar1*$^{+/fl\text{-}mZ\alpha}$ mice were further crossed with *Pgk-Cre* mice provided by Samira Lakhal-Littleton to produce *Adar1*$^{+/mZ\alpha}$ animals (Figure S1A). Primers F, R1, R2^{WT} and R2^{MUT} were used for genotyping; the sequences of these primers are listed in the Key Resources Table. PCR with primers F and R1 yielded a product of 357 bp for the WT *Adar1* allele and a 421 bp product for both '*fl-mZ*α' and '*mZ*α' alleles. PCR with primers F and R2^{WT} resulted in 1095 and 1158 bp products for the WT and '*fl-mZ*α' alleles, respectively, and no product for the '*mZ*α' allele. Finally, PCR with primers F and R2^{MUT} resulted in a 1158 bp product for the '*mZ*α' allele only.

Mavs$^{-/-}$ mice were a gift from C. Reis e Sousa and were originally from J. Tschopp (Michallet et al., 2008).

Cells

Lung fibroblasts and MEFs were grown in DMEM and BMMCs in RPMI, as described previously (Li et al., 2013; Maelfait et al., 2017). Media were supplemented with 10% heat-inactivated FCS and 2 mM L-glutamine; for BMMCs, 200 U/ml recombinant mouse GM-CSF (Peprotech) was added. MEFs were cultured at 3% oxygen.

METHOD DETAILS

RNA extraction and RT-qPCR

Organs collected from freshly killed mice (8-10 weeks of age) were snap frozen in liquid nitrogen immediately after dissection and stored at −80°C until further processing. Organs were homogenized with glass beads (425-600 μm, Sigma-Aldrich) in TRIzol (Thermo Fisher Scientific) using a FastPrep F120 instrument (Thermo Savant). RNA was extracted following the manufacturer's instructions and further purified using RNeasy Plus columns (QIAGEN) including a gDNA eliminator column step. cDNA synthesis was performed with SuperScript II reverse transcriptase (Thermo Fisher Scientific) with random hexamer (QIAGEN) or oligo $(dT)_{12\text{-}18}$ (Thermo Fisher Scientific) as primers. Gene-specific reverse transcription was primed with Taqman probes (Applied Biosystems). qPCR was done using Taqman Universal PCR Mix (Thermo Fisher Scientific) and Taqman probes. Alternatively, qPCR was performed using EXPRSS SYBR GreenER qPCR Supermix (Thermo Fisher Scientific) and DNA oligonucleotides (Sigma Aldrich). qPCR was performed on a QuantStudio 7 Flex real-time PCR system (Applied Biosystem). The qPCR probes and primers used in this study are listed in Table S2.

In vivo infection

WT and $Adar1^{mZ\alpha/mZ\alpha}$ mice were used at 8-10 weeks of age. Mice were intranasally inoculated with 50 μL A/X-31 (0.04 haemagglutination units (HAU)) diluted in viral growth medium (VGM; DMEM with 1% bovine serum albumin (Sigma-Aldrich A0336), 10 mM HEPES buffer, penicillin (100 U/ml) and streptomycin (100 μg/ml)) or mock infected with 50 μL VGM under light isoflurane anesthesia. Animals were assessed daily for weight loss and signs of disease. Mice reaching 20% weight loss were euthanised.

Median tissue culture infective dose ($TCID_{50}$) assay

Lungs from animals infected with IAV were snap frozen. Samples were then thawed and transferred into 250 μL ice cold VGM in Lysing Matrix D tubes (MP Biomedicals). Lungs were then homogenized using a FastPrep-24 Benchtop Tissue Homogenizer (MP Biomedicals) for 20 s at 4.0 m/s. Homogenized lungs were centrifuged for 10 minutes at 2,000 g to pellet debris. Virus containing supernatants were collected and stored at −80°C. 30,000 MDCK-SIAT1 cells were seeded per well in flat-bottom 96-well plates and allowed to adhere overnight at 37°C. Lung supernatants were diluted 100x in VGM and filtered through 0.22 μm syringe filters. MDCK-SIAT1 monolayers were washed with PBS and 50 μL of supernatants (diluted in a ½-log dilution series in VGM in quadruplicates) were added to infect the MDCK-SIAT1 cells for 1 hour at 37°C. Next, 150 μL VGM containing 1 μg/ml of TPCK trypsin (Thermo Scientific, 20233) was added and cells were incubated at 37°C for 48 hours. Cells were washed twice with PBS and then fixed with 100 μL of 10% formalin in PBS for 30 minutes at 4°C. Cells were then permeabilised with 50 μL of permeabilisation buffer (PBS, 20 mM glycine, 0.5% Triton X-100) for 20 minutes at room temperature. Cells were then washed twice with PBS and stained with 50 μL PBS containing 0.1% BSA and a chemically biotinylated anti-influenza NP antibody (2-8C, 1:250) as previously described (Powell et al., 2012) for 1 hour at room temperature. Cells were then washed twice with PBS and 50 μL of PBS containing Streptavidin, Alexa Fluor 647 Conjugate (Invitrogen S21374) at 1:500 dilution was added for 1 hour at room temperature. Cells were then washed and 100 μL of PBS containing 1% formalin were added to each well. Fluorescence intensity was measured on a ClarioStar Plate Reader (BMG Labtech). $TCID_{50}$ was calculated using the Reed & Muench method of cumulative percentage of positive and negative wells (Reed and Muench, 1938).

Western blot

Cells were lysed in RIPA buffer (50 mM Tris.HCl, pH7.4; 150 mM NaCl; 1% NP-40 (Sigma-Aldrich), 0.5% Deoxycholate, 0.1% SDS and Complete protease inhibitor (Roche)) at 4°C for 10 minutes. Protein lysates were then cleared by centrifugation at 13000 rpm for 10 minutes. Samples were mixed with NuPAGE SDS-PAGE sample loading buffer (ThermoFisher) containing 10% 2-mercaptoethanol. A primary antibody against ADAR1 was purchased from Santa Cruz (sc-73408). The antibody recognizing ISG15 was a gift from Klaus-Peter Knobeloch. HRP-coupled secondary antibodies were from GE Healthcare.

Flow cytometry

Lungs from 8-10 week old mice were dissected and mechanically disrupted using scissors before incubation in RPMI containing 1 μg/ml type II collagenase (Worthington Biochemical Corporation) and 40 U/ml DNase I (Sigma Aldrich) at 37°C for 60 minutes, with resuspension after 30 minutes to facilitate tissue dissociation. Cells were filtered through a 70 μm cell strainer (BD Falcon), rinsed with RPMI and pelleted at 400 x g for 5 minutes. The cell pellet was resuspended in 5 ml RBC lysis buffer (QIAGEN), incubated at room temperature for 5 minutes and then washed twice with 45 mL RPMI. Cells were resuspended in 500 μL FACS buffer (PBS containing 10% (v/v) FCS and 2 mM EDTA) and passed through a 70 μm cell strainer. Viable cells were counted using a haemocytometer. Cells were washed with PBS before incubation with LIVE/DEAD Fixable Aqua Dead Cell Stain (Invitrogen) diluted 1:200 in PBS for 30 minutes at 4°C. Cells were washed once with FACS buffer and then stained with surface antibodies diluted 1:200 (1:1000 for anti-MHCII-AF700) in Brilliant buffer (BD Biosciences) for 30 minutes. Cells were sorted directly into TRIzol-LS Reagent (Thermo Fisher Scientific) on BD FACSAria II and III machines (BD Biosciences). Alternatively, 1.5×10^6 cells were stained and analyzed using an Attune NxT Flow Cytometer (Thermo Fisher Scientific). Data were analyzed using FlowJo (v10.6.2).

CellPress
OPEN ACCESS

Magnetic cell fraction

Cells from lungs were prepared as described above. 10^7 cells were resuspended in 90 μL of MACS buffer (PBS containing 0.5% BSA and 2 mM EDTA) and then incubated with 10 μL of CD45 microbeads (Miltenyi) for 15 minutes. The mixture was then washed with MACS buffer and resuspend in 500 μL MACS buffer for magnetic separation on MACS LS columns (Miltenyi) according to the manufacturer's instructions. Cells were recovered from the flow-through (CD45-) and column (CD45+). 10% of cells were stained and analyzed by FACS to confirm purity. The remaining cells were pelleted, resuspend in TRIzol (ThermoFisher) and processed for RT-qPCR.

Generation of bone marrow chimeric animals

B6.SJL-CD45.1 mice were used as bone marrow recipients and were lethally irradiated twice (4.5 Gy for 300 s, separated by a ~3 hour rest). Mice were then injected intravenously with bone marrow from either WT (CD45.2) or *Adar1*$^{mZ\alpha/mZ\alpha}$ mice. Recipient mice received antibiotics (0.16 mg/mL, Enrofloxacin (Baytril), Bayer Corporation) in drinking water for four weeks following irradiation and were rested for > 8 weeks before tissue collection.

ELISA

Mouse IL-6 was quantified by uncoated ELISA Kit (ThermoFisher) according to manufacturer's instruction.

RNA-seq and data processing

Stranded Illumina sequencing libraries were prepared with the RNA-Seq Ribozero kit from isolated RNAs and submitted for PE150 sequencing using an Illumina NovaSeq6000 machine, yielding ~100M reads per sample. Sequencing data was processed using a Nextflow v20.07 (Di Tommaso et al., 2017) pipeline automating quality control using FastQC v0.11.8 (bioinformatics.babraham.ac.uk/projects/fastqc/), quality and adaptor trimming using cutadapt v1.18 (Martin, 2011), contaminant detection using screen.sh (within BBMap v36.20, sourceforge.net/projects/bbmap/), strand-aware alignment using HISAT2 v2.1.0 (Kim et al., 2019) and STAR v2.7.1a (Dobin et al., 2013), post-alignment quality-assurance using 'gene body coverage', 'transcript integrity', and 'inner distance' metrics from RSeQC v2.6.4 (Wang et al., 2012), and strand-specific counting of uniquely-mapping reads using featureCounts (within Subread v1.6.4, (Liao et al., 2014)) against Ensembl GRCm38.100 annotations. Additional, unstranded counts were obtained with featureCounts against a database of repetitive elements previously prepared for GRCm38 (Attig et al., 2017) using reads unassigned to features during the previous step.

Differential expression analysis

Downstream differential expression analysis was conducted using counts obtained for STAR read mappings using DESeq2 (Love et al., 2014) (v1.22.1) within R (v4.0.2). Gene ontology analysis was performed using goseq (Young et al., 2010) (1.34.1). Heatmaps were generated using the pheatmap package (v1.0.12).

Detection of A-to-I editing

A Python 3.8 program, edIted (github.com/A-N-Other/pedestal, commit 2c55bf3), was produced to identify editing sites. edIted performs stranded assessments of RNA editing from samtools mpileup (Li, 2011) data, building on the Dirichlet-based models implemented in ACCUSA2 and JACUSA (Piechotta and Dieterich, 2013; Piechotta et al., 2017). When run with test data alone, edIted runs in 'detect' mode, finding base modifications by comparing the goodness of fit of Dirchlet models of the base error (derived from the Phred quality data in the mpileup input) and the background sequencing error to the base frequencies recorded at a specific position. With an additional control dataset, edIted runs in 'differential' mode, performing the above analysis to determine significantly edited sites before additionally testing for differential editing by comparing the goodness of fit of Dirichlet models of the base error from the test and control datasets to their own and each other's base frequencies. When biological replicates are provided, edIted adjusts the reported Z scores to reflect the proportion of test dataset samples displaying editing. edIted was run in both modes with samtools mpileup files (supplemented with TS tag metadata) separately for HISAT2 and STAR alignments of the data with the flags '–min_depth 5–min_alt_depth 2–min_edited 0.01–max_edited 0.9–z_score 2.58'. For differential analyses the '–min_fold 2′ flag was used and, where considering biological replicates the '–reps 3′ flag, such that editing is required in all three samples. All analyses were conducted supplying BED files of ENCODE blacklisted regions (Amemiya et al., 2019) and known splice sites (regions set to splice site ± 2 nts) to the '–blacklist' flag. Sites that were found in common between the HISAT2- and STAR-mapped data were retained for further analysis.

Analysis of A-to-I editing sites

Sites obtained from edIted were assigned to genomic features using annotatr v1.16 within R (Cavalcante and Sartor, 2017). Assessments of editing enrichment within repetitive elements were conducted using regioneR v1.22 within R (Gel et al., 2016) using randomization-based permutation tests with 100 bootstraps. Assessment of distance to neighboring inverted SINE super-family (B1, B2, B3, B4) members was conducted with bedtools v2.29.2 closest (Quinlan and Hall, 2010) using the '-io -S' flags. Outputs and the detailed statistics were produced with GraphPad Prism v8. Regions ± 500 nts surrounding editing sites (total length of 1001 nts) were extracted using samtools and used to calculate per-position fractional GC content centered around the edited position and, separately, as input to Zhunt3 using the settings 'windowsize = 12 minsize = 12 maxsize = 12′ (Champ et al., 2004; Ho et al., 1986). Scores from

Zhunt were log transformed for plotting. The SIBZ algorithm for finding Z structured regions was run using the qsidd program within the SIST software package (Zhabinskaya and Benham, 2011) by passing the '-Z' runtime flag. Results were filtered for regions with > 90% probability of exhibiting Z structure and runs of immediately adjacent sites concatenated with bedtools. Assessment of distances between editing sites and regions identified by SIBZ were conducted with bedtools closest.

QUANTIFICATION AND STATISTICAL ANALYSIS

All experiments were performed three times or more independently under similar conditions, unless specified otherwise in figure legends. Statistical significance was calculated as described in the figure legends; $p < 0.05$ was considered significant. GraphPad Prism 8 software was used to generate graphs and to perform statistical analysis.

Immunity, Volume *54*

Supplemental information

Adenosine-to-inosine editing of endogenous Z-form RNA by the deaminase ADAR1 prevents spontaneous MAVS-dependent type I interferon responses

Qiannan Tang, Rachel E. Rigby, George R. Young, Astrid Korning Hvidt, Tanja Davis, Tiong Kit Tan, Anne Bridgeman, Alain R. Townsend, George Kassiotis, and Jan Rehwinkel

Figure S1.

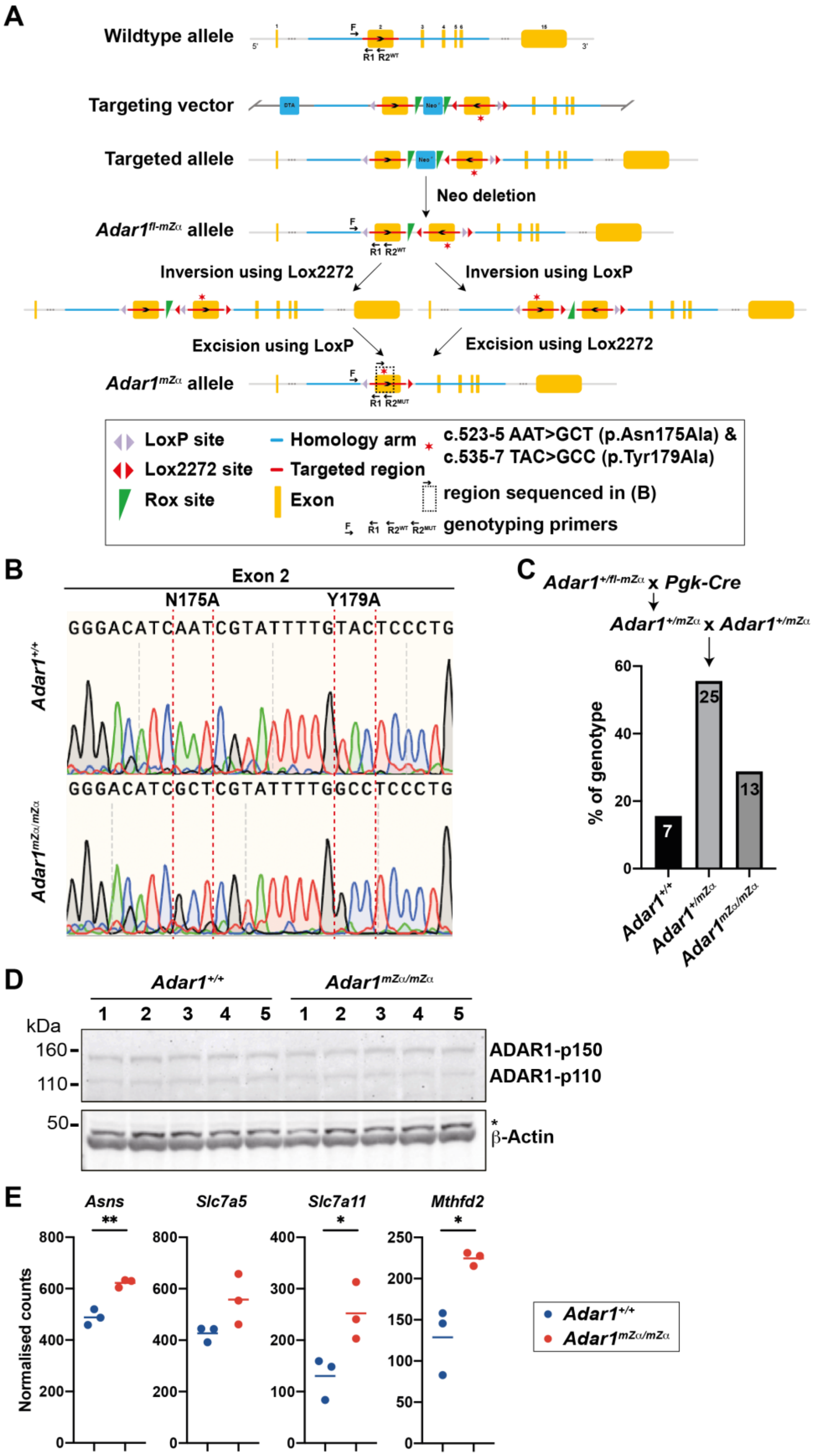

A
Wildtype allele
Targeting vector
Targeted allele
Neo deletion
Adar1fl-mZα allele
Inversion using Lox2272
Inversion using LoxP
Excision using LoxP
Excision using Lox2272
Adar1mZα allele
LoxP site
Lox2272 site
Rox site
Homology arm
Targeted region
Exon
c.523-5 AAT>GCT (p.Asn175Ala) &
c.535-7 TAC>GCC (p.Tyr179Ala)
region sequenced in (B)
genotyping primers
B
Exon 2
N175A
Y179A
GGGACATCAATCGTATTTTGTACTCCCTG
GGGACATCGCTCGTATTTTGGCCTCCCTG
Adar1+/+
Adar1mZα/mZα
C
Adar1+/fl-mZα x Pgk-Cre
Adar1+/mZα x Adar1+/mZα
% of genotype
Adar1+/+
Adar1+/mZα
Adar1mZα/mZα
D
Adar1+/+
Adar1mZα/mZα
kDa
ADAR1-p150
ADAR1-p110
β-Actin
E
Asns
Slc7a5
Slc7a11
Mthfd2
Normalised counts
Adar1+/+
Adar1mZα/mZα

Figure S1, related to Figures 1 and 2. Generation of *Adar1*$^{mZ\alpha/mZ\alpha}$ animals.

A. Schematic representation of the *Adar1* WT allele, targeting vector, targeted allele, *Adar1*$^{fl\text{-}mZ\alpha}$ allele and the two-step Cre-mediated recombination process that resulted in the *Adar1*$^{mZ\alpha}$ allele. Please see text for details.

B. Genomic DNA was prepared from WT and *Adar1*$^{mZ\alpha/mZ\alpha}$ animals and the mutated region in exon 2 was sequenced.

C. *Adar1*$^{+/fl\text{-}mZ\alpha}$ mice were bred with the *Pgk-Cre* line. *Adar*$^{+/mZ\alpha}$ offspring were then mated to generate *Adar1*$^{mZ\alpha/mZ\alpha}$ animals. The numbers and percentages of animals obtained with the indicated genotypes are shown.

D. BMMCs were grown from bone marrow from five mice of the indicated genotypes. Protein extracts were used for western blot with α-ADAR1 antibody. β-Actin served as a loading control. *, non-specific band

E. Normalised read counts for the indicated transcripts from lung RNA sequencing (see Figure 2) are shown.

Figure S2.

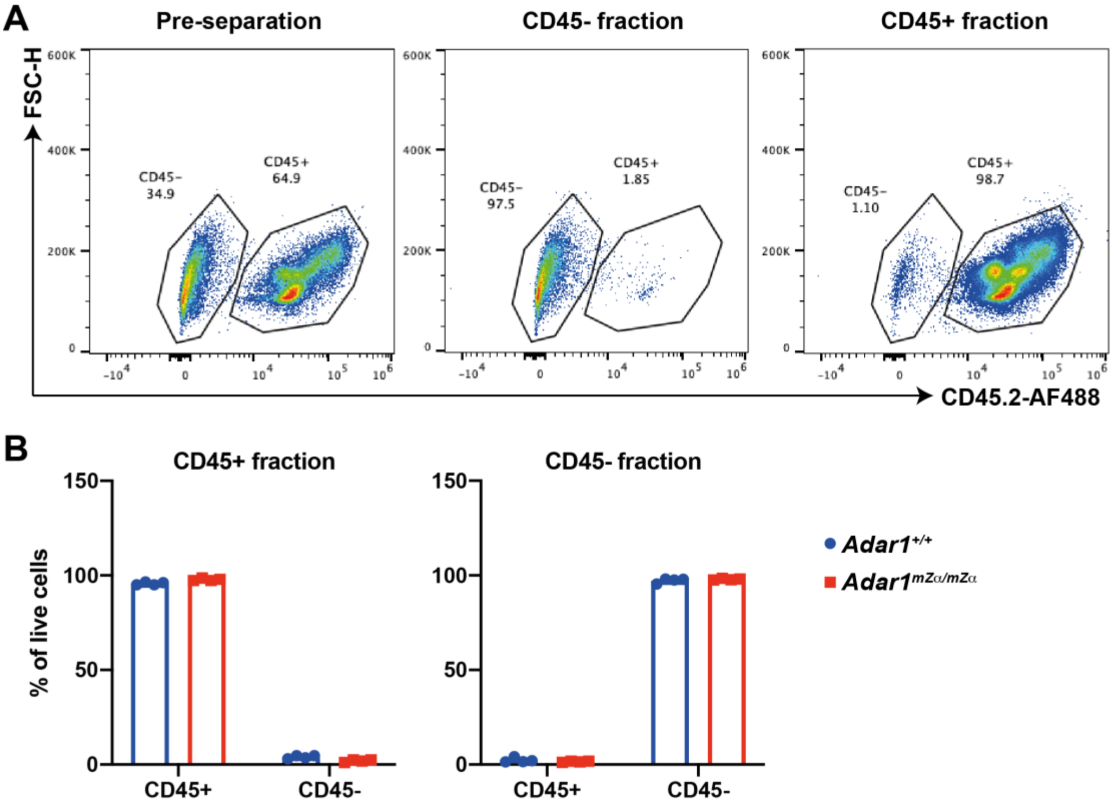

Figure S2, related to Figure 3. MACS separation of lung cells.

A. Cell surface levels of CD45 were analysed by flow cytometry in single cell suspensions obtained from lung tissue before MACS (left) and in CD45- and CD45+ cell fractions obtained after MACS (middle and right). Data are from a representative WT animal.

B. The percentage of CD45-expressing cells is shown for CD45+ and CD45- MACS fractions. Data points represent individual animals (n=4) from a representative experiment and bars indicate the mean.

Figure S3.

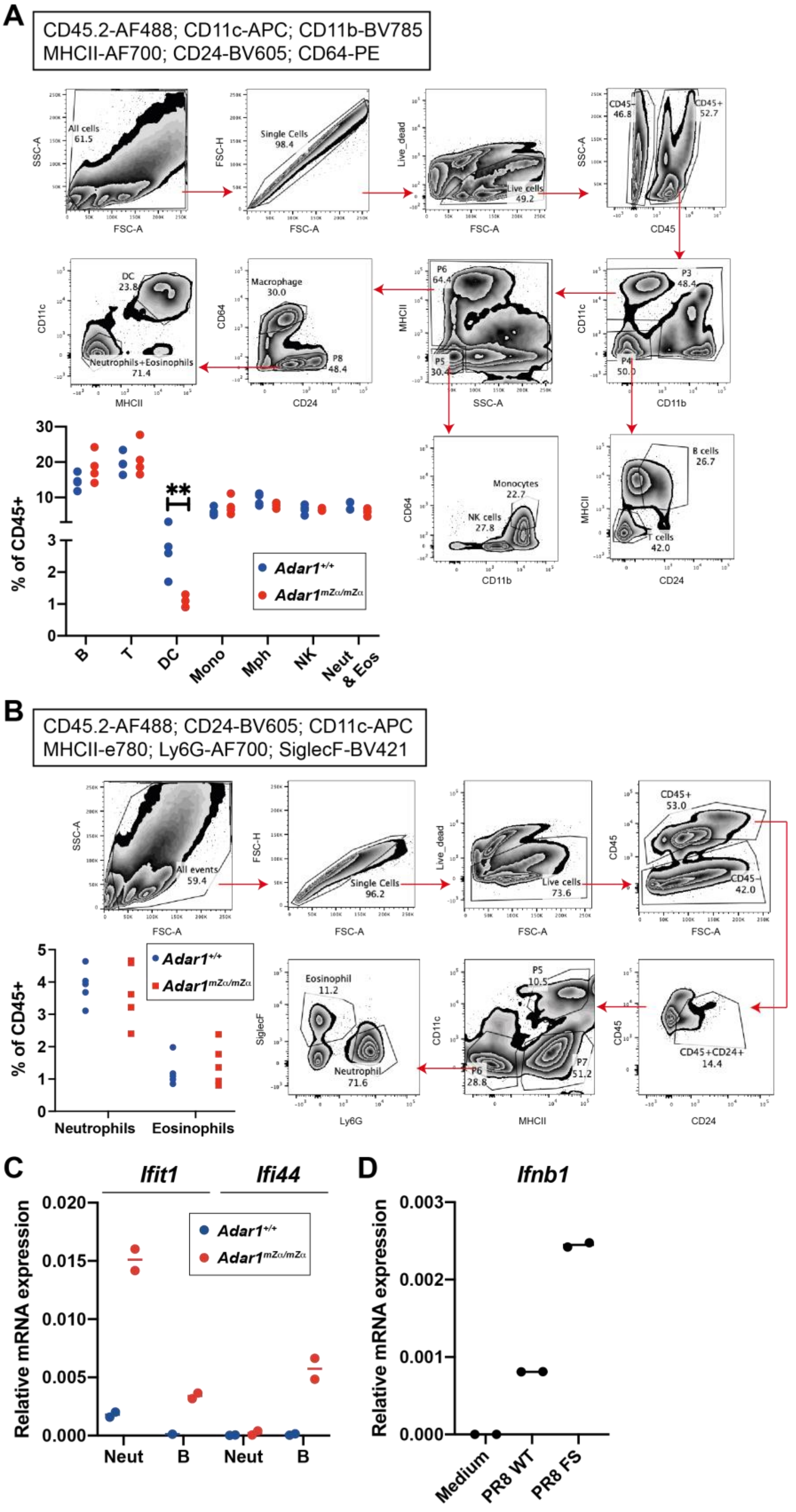
A
CD45.2-AF488; CD11c-APC; CD11b-BV785
MHCII-AF700; CD24-BV605; CD64-PE
All cells 61.5
Single Cells 98.4
Live cells 49.2
CD45- 46.8
CD45+ 52.7
DC 23.8
Neutrophils+Eosinophils 71.4
Macrophage 30.0
P8 48.4
P6 64.4
P5 30.4
P3 48.4
P4 50.0
Monocytes 22.7
NK cells 27.8
B cells 26.7
T cells 42.0
% of CD45+
B
T
DC
Mono
Mph
NK
Neut & Eos
Adar1+/+
Adar1mZα/mZα
B
CD45.2-AF488; CD24-BV605; CD11c-APC
MHCII-e780; Ly6G-AF700; SiglecF-BV421
All events 59.4
Single Cells 96.2
Live cells 73.6
CD45+ 53.0
CD45- 42.0
CD45+CD24+ 14.4
P5 10.5
P7 51.2
P6 28.8
Eosinophil 11.2
Neutrophil 71.6
Neutrophils
Eosinophils
C
Ifit1
Ifi44
Relative mRNA expression
Neut
B
D
Ifnb1
Medium
PR8 WT
PR8 FS

Figure S3, related to Figure 4A and 4B. Gating strategy for sorting of haematopoietic lung cells.

A-B. Two staining panels were used to identify and isolate haematopoietic cell populations by FACS. Panel (A) is related to Figure 4A and panel (B) to Figure 4B. Antibodies and conjugated fluorophores are shown in boxes. Gating strategies are shown for a representative WT (A) and *Adar1*$^{mZ\alpha/mZ\alpha}$ (B) animal. Bar graphs show the proportion of each cell population as a percentage of CD45+ cells. Each dot represents an individual mouse and data from two independent experiments were pooled (**p<0.01, unpaired t test).

C. Neutrophils and B cells were sorted from the lungs of WT and *Adar1*$^{mZ\alpha/mZ\alpha}$ mice. RNA samples were reverse transcribed with gene specific primers for *Ifit1*, *Ifi44* and *Ifnb1*, followed by qPCR. *Ifnb1* mRNA levels were undetectable.

D. RNA extracted from whole lungs of WT mice 48 hours after intranasal infection with 50,000 pfu of IAV PR8 or of a mutant PR8 virus (PR8 FS; known to induce higher levels of *Ifnb1*) were analysed as in (C).

In (C-D), mRNA levels are shown relative to *Actb*. Each dot represents samples from an individual mouse.

Figure S4.

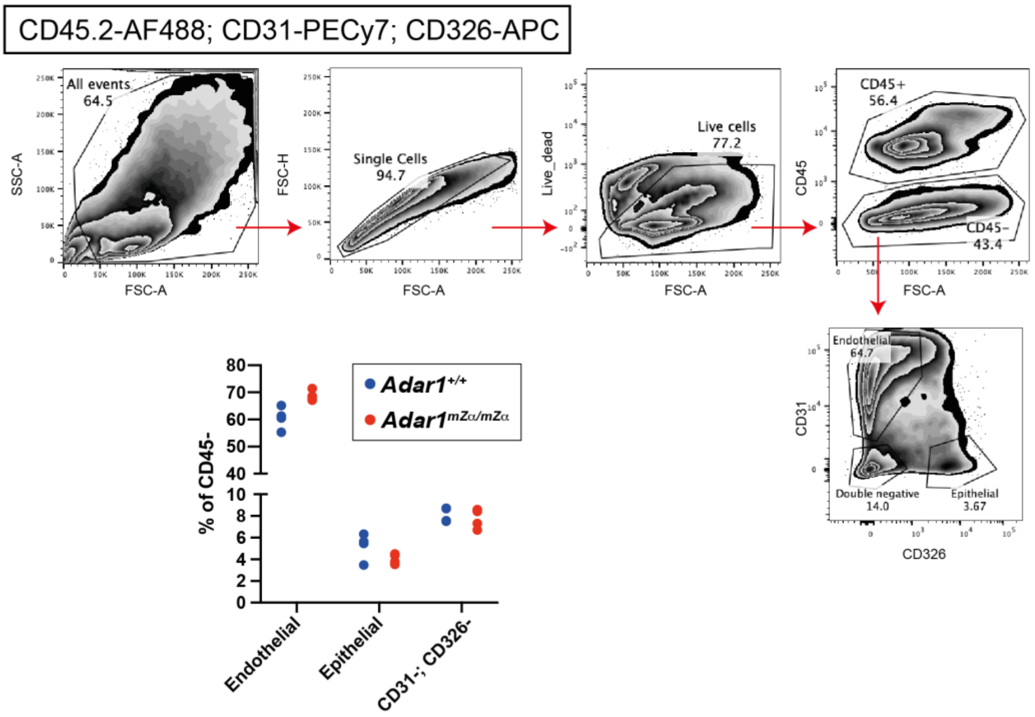

Figure S4, related to Figure 4C. Gating strategy for sorting of stromal lung cells. The staining panel used to identify and isolate non-haematopoietic cell populations by FACS is shown. Antibodies and conjugated fluorophores are shown in the box. The gating strategy is shown for a representative WT animal. The bar graph shows the proportion of each cell population as a percentage of CD45- cells. Each dot represents an individual mouse.

Figure S5.

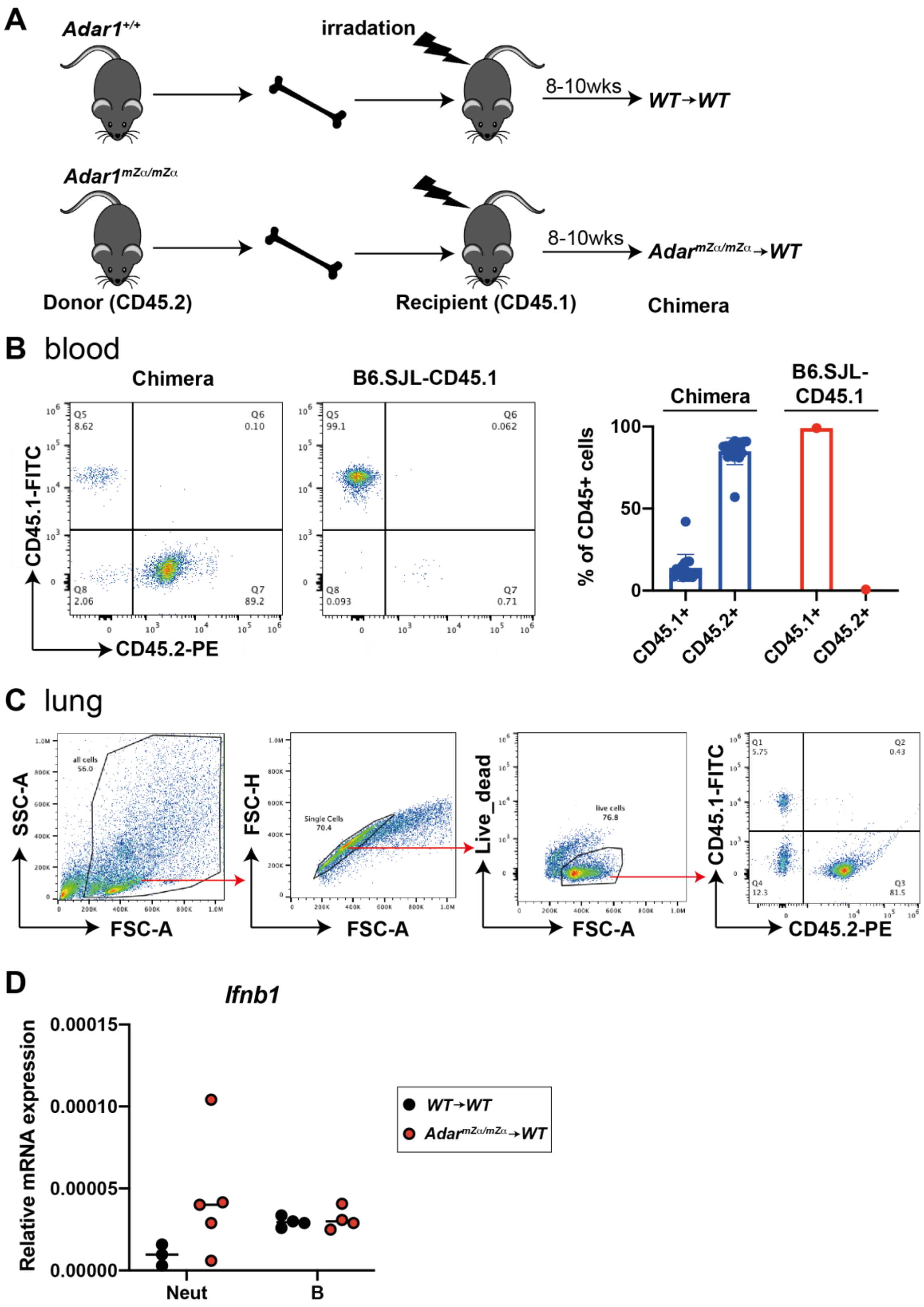
A
Adar1+/+
irradiation
8-10wks
WT→WT
Adar1mZα/mZα
8-10wks
AdarmZα/mZα→WT
Donor (CD45.2)
Recipient (CD45.1)
Chimera
B blood
Chimera
B6.SJL-CD45.1
CD45.1-FITC
CD45.2-PE
% of CD45+ cells
CD45.1+
CD45.2+
C lung
SSC-A
FSC-A
FSC-H
Live_dead
all cells
Single Cells
live cells
D
Ifnb1
Relative mRNA expression
Neut
B
WT→WT
AdarmZα/mZα→WT

Figure S5, related to Figure 4D. Analysis of BM chimeras.

A. Schematic representation of the generation of BM chimeric animals.

B. White blood cells from BM chimeric mice and, as control, from an untreated B6.SJL-CD45.1 animal, were analysed by FACS. Cells were gated on single, live cells. Representative FACS plots from a WT→WT animal (left) and pooled data from two independent experiments involving a total seven WT→WT and eight *Adar1*$^{mZ\alpha/mZ\alpha}$→WT BM chimeric animals (right) are shown. Bars show the mean and error bars represent SD.

C. Lung cells from BM chimeric mice were analysed by FACS. Data from a representative WT→WT animal are shown.

D. Neutrophil and B cell RNA samples obtained from the lungs of BM chimeric animals were tested by conventional RT qPCR for *Ifnb1* transcript levels. mRNA levels are shown relative to *Actb*. Each dot represents samples from an individual mouse.

Figure S6.

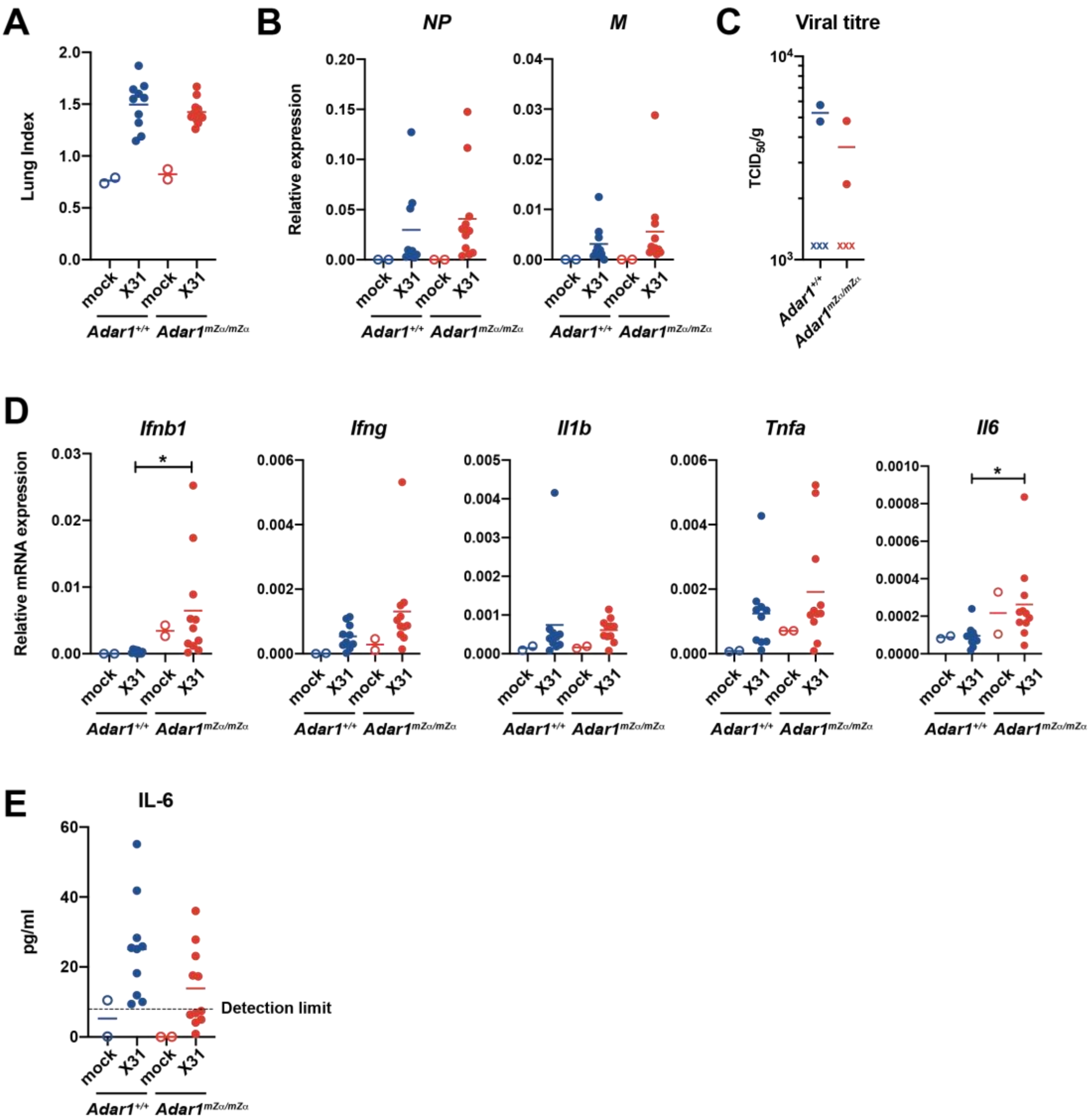
A
B
C
D
E
Lung Index
NP
M
Relative expression
Viral titre
TCID50/g
Ifnb1
Ifng
Il1b
Tnfa
Il6
Relative mRNA expression
IL-6
pg/ml
Detection limit
mock
X31
Adar1+/+
Adar1mZα/mZα
xxx

Figure S6. Analysis of WT and *Adar1*$^{mZ\alpha/mZ\alpha}$ mice on day 7 after IAV infection. Related to Figure 5.

A-E. WT or *Adar1*$^{mZ\alpha/mZ\alpha}$ mice were infected intranasally with 0.04 HAU of IAV strain A/X-31. On day 7 post infection, lungs and sera were collected.

A. The 'lung index' was calculated (lung weight/body weight x100).

B. Levels of the viral *NP* and *M* transcripts were analysed by RT-qPCR in RNA samples extracted from total lung. Data are shown relative to *Actb* (*NP*) or *Gapdh* (*M*).

C. Lung viral titres were determined in samples from infected animals by $TCID_{50}$ analysis and were normalised to lung weight. Crosses indicate samples without detectable viral titre.

D. Levels of the indicated mRNAs were determined as in (B).

E. Serum IL-6 concentrations were analysed by ELISA.

In (A-B and D-E), pooled data from two independent experiments (mock infected: n=2 mice per genotype; A/X-31-infected: n=10 WT and n=11 *Adar1*$^{mZ\alpha/mZ\alpha}$ mice) are shown. In (C), data from one experiment with n=5 infected mice per genotype are shown. Each dot represents an individual mouse and the mean is indicated (*p<0.05, unpaired t test).

Figure S7.

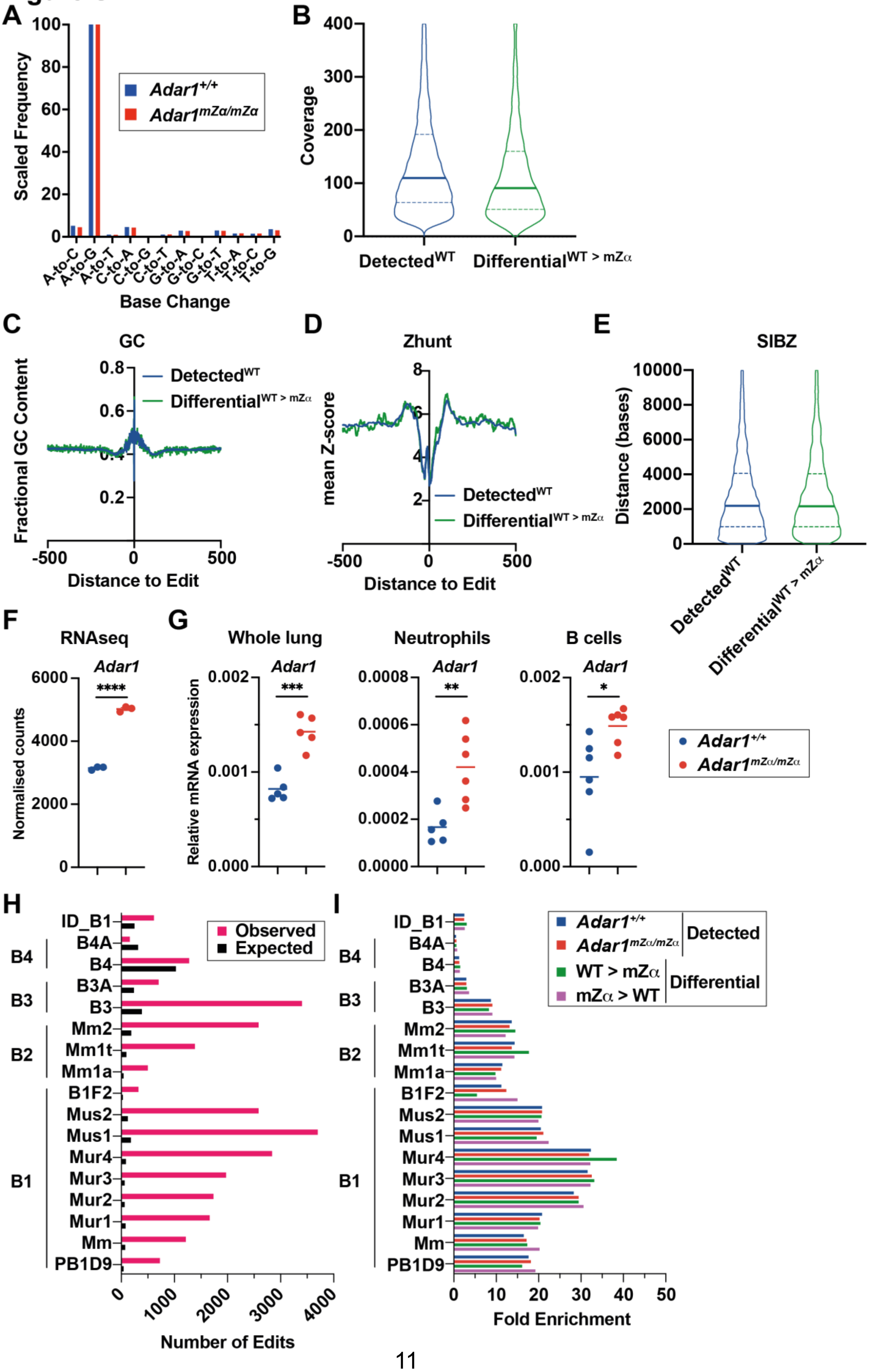
A
Scaled Frequency
Adar1+/+
Adar1mZα/mZα
A-to-C A-to-G A-to-T C-to-A C-to-G C-to-T G-to-A G-to-C G-to-T T-to-A T-to-C T-to-G
Base Change
B
Coverage
DetectedWT
DifferentialWT > mZα
C
GC
Fractional GC Content
Distance to Edit
D
Zhunt
mean Z-score
Distance to Edit
E
SIBZ
Distance (bases)
F
RNAseq
Adar1
Normalised counts
G
Whole lung
Neutrophils
B cells
Relative mRNA expression
H
Observed
Expected
Number of Edits
ID_B1 B4A B4 B3A B3 Mm2 Mm1t Mm1a B1F2 Mus2 Mus1 Mur4 Mur3 Mur2 Mur1 Mm PB1D9
I
Detected
Differential
WT > mZα
mZα > WT
Fold Enrichment

Figure S7. Analysis of RNA editing. Related to Figure 7.

A. Differences between the reference genome and RNAseq data were analysed for all possible base changes. The frequency of A-to-G changes was set to 100 for WT and *Adar1*$^{mZ\alpha/mZ\alpha}$ lung samples.

B. Sequence coverage for 'detected^{WT}' and 'differential$^{WT>mZ\alpha}$' sites is shown in violin plots. Solid horizontal lines show the median and dotted lines indicate quartiles.

C. Nucleotide composition was analysed in 1001 nt windows centred on editing sites detected in WT mice or on 'differential$^{WT>mZ\alpha}$' sites. The fractional GC content is shown.

D. The Zhunt algorithm was used to calculate the propensity of sequences within 500 nt from editing sites to form the Z conformation. Log10 transformed mean z-scores are shown for 'detected^{WT}' and 'differential$^{WT>mZ\alpha}$' sites.

E. The distances of editing sites to genomic regions predicted by SIBZ to form the Z-conformation is shown in violin plots. Solid horizontal lines show the median and dotted lines indicate quartiles.

F. Normalised *Adar1* read counts from lung RNA sequencing (see Figure 2) are shown.

G. *Adar1* mRNA levels were analysed by RT-qPCR in RNA samples extracted from whole lungs (left) or from FACS-enriched lung neutrophils (middle) or lung B cells (right) of WT and *Adar1*$^{mZ\alpha/mZ\alpha}$ animals. Data are shown relative to *Actb*.

H. Sub-families of SINEs were analysed as in Figure 7D, but are plotted to include those where observed or expected values exceeded 250.

I. Fold enrichments of editing events were calculated relative to expected numbers of edits for SINE sub-families using editing events detected in WT or *Adar1*$^{mZ\alpha/mZ\alpha}$ samples and differentially edited sites.

In (F) and (G), each dot represents an individual mouse and horizontal lines show the mean. In (F), lungs from 3 mice per genotype were sequenced. In (G),

samples from 5-6 mice per genotype were analysed. Data were analysed by unpaired t test (****$p<0.0001$, ***$p<0.001$, **$p<0.01$, *$p<0.05$).

Table S2. qPCR probes and primers. Related to STAR Methods.

**Taqman Probes**	**Assay Probe ID**
Ifnb1	Mm00439552_s1
Ifng	Mm01168134_m1
Tnfa	Mm00443258_m1
Il1b	Mm00434228_m1
Il6	Mm00446191_m1
Ifit1	Mm00515153_m1
Ifit2	Mm00492606_m1
Ifi44	Mm00505670_m1
Isg15	Mm01705338_s1
Isg20	Mm00469585_m1
Zbp1	Mm01247052_m1
Oas1a	Mm00836412_m1
NP	Custom probe based on NC_002019.1<br>Assay ID:AIX02UC
Actin	Mm02619580_g1
Gapdh	Mm99999915_g1 (4352932E)
**SYBR Green oligos**	**Sequence**
M	F: 5'-CTTCTAACCGAGGTCGAAACGTA<br>R: 5'-GGTGACAGGATTGGTCTTGTCTTTA<br>from: Shin et al. 2013, Virology Journal 10:303
Gapdh	F: 5'-CATGGCCTTCCGTGTTCCTA<br>R: 5'-CCTGCTTCACCACCTTCTTGAT<br>from: Tsujita et al. 2006, PNAS 103:11946
